# Phylogenetic and geographical analysis of a retrovirus during the early stages of endogenous adaptation and exogenous spread in a new host

**DOI:** 10.1111/mec.15735

**Published:** 2020-12-30

**Authors:** Bonnie L. Quigley, Faye Wedrowicz, Fiona Hogan, Peter Timms

**Affiliations:** ^1^ Genecology Research Centre University of the Sunshine Coast Sippy Downs QLD Australia; ^2^ School of Science, Psychology and Sport Federation University Australia Churchill Vic. Australia

**Keywords:** endogenous, exogenous, gammaretrovirus, koala, KoRV, Phascolarctos cinereus, retrovirus

## Abstract

Most retroviral endogenization and host adaptation happened in the distant past, with the opportunity to study these processes as they occurred lost to time. An exception exists with the discovery that koala retrovirus (KoRV) has recently begun its endogenization into the koala (*Phascolarctos cinereus*) genome. What makes this opportunity remarkable is the fact that Northern Australian koalas appear to be undergoing endogenization with one KoRV subtype (KoRV‐A), while all subtypes (KoRV‐A‐I) coexist exogenously, and Southern Australian koalas appear to carry all KoRV subtypes as an exogenous virus. To understand the distribution and relationship of all KoRV variants in koalas, the proviral KoRV envelope gene receptor binding domain was assessed across the koala's natural range. Examination of KoRV subtype‐specific proviral copy numbers per cell found that KoRV‐A proviral integration levels were consistent with endogenous incorporation in Northern Australia (southeast Queensland and northeast New South Wales) while revealing lower levels of KoRV‐A proviral integration (suggestive of exogenous incorporation) in southern regions (southeast New South Wales and Victoria). Phylogeographical analysis indicated that several major KoRV‐A variants were distributed uniformly across the country, while non‐KoRV‐A variants appeared to have undergone lineage diversification in geographically distinct regions. Further analysis of the major KoRV‐A variants revealed a distinct shift in variant proportions in southeast New South Wales, suggesting this as the geographical region where KoRV‐A transitions from being predominantly endogenous to exogenous in Australian koalas. Collectively, these findings advance both our understanding of KoRV in koalas and of retroviral endogenization and diversification in general.

## Abstract 2

see also the Perspective by Elliott S. Chiu and Roderick B. Gagne.

## 1 INTRODUCTION

In 2000, a novel gammaretrovirus, termed koala retrovirus (KoRV), was identified in koalas (*Phascolarctos cinereus*; Hanger et al., 2000). This discovery was exciting, not only for koala researchers, but also for the general virology community. This was because this new retrovirus was found to be in the active process of endogenizing (incorporating into germline DNA) within the koala population (Tarlinton et al., 2006). Sequence analysis of the viral long terminal repeats (LTRs) indicated that this process began no more than 22,200–49,900 years ago (although a much more recent start to endogenization is also possible; Ishida et al., 2015). This is in contrast to the endogenization of many other known mammalian retroviruses, some of which occurred millions of years ago (Stoye, 2012). Studies have determined that KoRV currently exists as both an endogenous and an exogenous virus, in different koala subpopulations (Xu & Eiden, 2015). This complex state of host and retrovirus interaction has created a very useful natural model in which to study retroviral evolution and endogenization in real time.

KoRV is related to several gammaretroviruses, including gibbon ape leukaemia virus (GALV; Hanger et al., 2000), *Melomys burtoni* retrovirus (MbRV; Simmons et al., 2014), *Melomys* woolly monkey virus (MelWMV; Alfano et al., 2016) and hervey pteropid gammaretrovirus (HPG; Hayward et al., 2020). KoRV is currently divided into three major clades and nine subtypes, based on the envelope (*env*) gene receptor binding domain (RBD; Chappell et al., 2017; Hanger et al., 2000; Shojima et al., 2013; Xu et al., 2013). KoRV‐A is the most prevalent clade and subtype across Australia, found endogenously and exogenously in northern koalas (Chappell et al., 2017; Hanger et al., 2000; Tarlinton et al., 2006) and is believed to be predominantly exogenous in southern koalas (Legione et al., 2017; Simmons et al., 2012). Studies examining KoRV endogenization agree that KoRV‐A integration into the koala genome is still in its active early stages, with multiple independent integration events detected in each group of koalas studied (Cui et al., 2016; Ishida et al., 2015; Tsangaras et al., 2014). KoRV‐B was the second distinct clade and subtype to be identified. KoRV‐B appears to be exogenously spread, and its proviral detection has been associated with increased chlamydial disease and malignant neoplasms in koalas (Chappell et al., 2017; Quigley et al., 2018; Waugh et al., 2017). Finally, KoRV‐C to ‐I are the subtypes most recently recognized and form the third major clade, with KoRV‐D and KoRV‐F commonly reported as the dominant subtypes within this group (Chappell et al., 2017; Quigley et al., 2018; Sarker et al., 2019). The subtypes within the KoRV‐D/F/other clade appear exogenously transmitted and, as yet, their proviral detection has not been linked to adverse health outcomes in koalas. Sequencing of the first complete koala genome revealed intact proviral sequences for KoRV‐A and KoRV‐B, but only defective proviral sequences (missing large portions of the *gag* and *pol* genes) for KoRV‐D and KoRV‐E in this northern koala (Hobbs et al., 2017). Additionally, data from Southern Australia suggest that defective KoRV, at either the proviral or the expression level, may be the predominant form of KoRV in koalas from these southern regions (Sarker et al., 2020). Collectively, our knowledge of KoRV proviral diversity, both by subtype and by defective status, as well as endogenization pattern, is still in its infancy.

The prevalence of KoRV in koalas across Australia has been examined in two national surveys (Simmons et al., 2012; Tarlinton et al., 2006). These surveys were conducted before the different subtypes of KoRV were recognized, so results were limited to total KoRV provirus presence or absence. In 2006, an initial survey found central Queensland (CEQLD) and southeast Queensland (SEQLD) koalas (in the north) had 100% KoRV prevalence (*n* = 98) while Victoria (VIC) mainland and island koalas (in the south) had 60% (*n* = 5) and 29% (*n* = 17) KoRV prevalence, respectively (Tarlinton et al., 2006). Kangaroo Island, South Australia (SA; also in the south) had no KoRV detected (*n* = 26; Tarlinton et al., 2006). In 2012, a larger survey found similar results, with koalas from CEQLD, SEQLD and northeast New South Wales (NENSW; all northern regions) having a 100% prevalence for KoRV (*n* = 377) while koalas from VIC had 73% KoRV prevalence on the mainland (*n* = 89) and 27% prevalence on the nearby islands (*n* = 80; Simmons et al., 2012). This second study now found Kangaroo Island, SA, to have a KoRV prevalence of 15% (*n* = 162; albeit from a different geographical location as the 2006 study; Simmons et al., 2012). While these studies indicate that KoRV is ubiquitous in northern koalas and increasing in prevalence in southern koalas, detailed information about KoRV subtype composition and phylogenetic relatedness across Australia is still lacking.

Determining proviral copies per cell is a key measurement when investigating the endogenization state of a retrovirus. For KoRV, several approaches have been taken to estimate KoRV proviral copies per cell, including quantification of KoRV *pol* gene copies per nanogram of sample DNA (Simmons et al., 2012), quantification of KoRV *pol* or *env* gene targets in comparison to copies of the koala β‐actin gene (Sarker et al., 2020; Wedrowicz et al., 2016), and direct counting of proviral sequences in the koala genome (Hobbs et al., 2017). These studies have detected 5.47 × 10^1^ to 1.65 × 10^2^ KoRV proviral copies per cell from Queensland (QLD) koalas (Sarker et al., 2020; Simmons et al., 2012), with approximately half of these provirus being KoRV‐A (Hobbs et al., 2017). From NENSW, KoRV‐A proviral copies per cells have been detected at 4.0 × 10^0^ copies per cell (Wedrowicz et al., 2016). Finally, VIC koalas have been reported to carry 9.0 × 10^−2^ to 1.29 × 10^−4^ KoRV proviral copies per cell (Simmons et al., 2012; Wedrowicz et al., 2016). These studies indicate that KoRV provirus generally appears at levels typical for endogenous virus in QLD and NSW koalas and exogenous virus in VIC koalas.

Our ability to genetically profile KoRV has advanced considerably in recent years. A molecular test now exists to simultaneously detect all KoRV proviral subtypes present in a sample (Chappell et al., 2017). Similar to bacterial microbiome study methods, this KoRV method has the additional advantage of also capturing sequence data from the RBD of the *env* gene, allowing for detailed analysis of KoRV complexity and evolutionary relatedness (Chappell et al., 2017; Quigley et al., 2018, 2019; Sarker et al., 2019). The study presented here uses this detailed molecular KoRV profiling technique to phylogeographically profile KoRV provirus *env* gene sequence across Australia. Together with KoRV proviral copy number per cell measurements, KoRV‐A variant analysis was used to investigate whether a geographical region in Australia could be identified where KoRV‐A appears to transition from being predominantly endogenous to predominantly exogenous in koalas. Additionally, phylogeographical analysis examined non‐KoRV‐A variant patterns of dispersal and diversification.

## 2 MATERIALS AND METHODS

### 2.1 Sample collection

Koala samples were collected from animals across their natural range, including SEQLD, NENSW, southeast New South Wales (SENSW) and VIC. It is well documented that koalas in SA have been translocated to this state from VIC and QLD koala populations (Lindsay, 1950; Martin, 1989; Wedrowicz et al., 2017). As it would be difficult to deconvolute KoRV evolution and spread in SA koalas with this recent human intervention, koalas from this state were deliberately excluded from this study.

Samples from SEQLD koalas (*n* = 10 matched blood/urogenital swab/scat samples, plus *n* = 14 additional blood samples) were collected as part of an ongoing population‐wide health management programme. All procedures were approved by the University of the Sunshine Coast Animal Ethics Committee (Animal ethics number AN/A/13/80) and by the Queensland Government (Scientific Purposes Permit, WISP11532912). All collections were performed in accordance with relevant guidelines and regulations. Blood and urogenital swabs were collected from koalas under general anaesthesia during veterinary examinations. Scats were collected from the koala transport cage after the koala was transported to the veterinary facility for examination. Koala scats from NENSW, SENSW and VIC were collected as part of a previous study where individuals were identified using microsatellite genotyping (Wedrowicz et al., 2018). The NENSW, SENSW and VIC sample set included samples from both ear clips (*n* = 48) and scats (*n* = 229).

### 2.2 DNA extraction

DNA was isolated from scat samples using the QIAamp DNA Stool Mini Kit (Qiagen) or the AxyPrep MAG Soil, Stool, and Water DNA Kit (Axygen) as previously described (Wedrowicz et al., 2013, 2018). DNA was isolated from ear clips using the DNeasy Blood & Tissue Kit (Qiagen) following the manufacturer's protocol. Matched blood, urogenital swab and scat samples were all extracted using the QIAamp DNA Mini Kit (Qiagen) following the manufacturer's protocol for each sample type.

### 2.3 KoRV *env* proviral amplicon generation and sequencing

KoRV *env* amplicons spanning the RBD of the *env* gene (positions 22–514 of the KoRV‐A *env* gene) were generated with the universal *env* primers env22.F (TCGTCGGCAGCGTCAGATGTGTATAAGAGACA GGCTTCTCATCTCAAACCCGCGCC) and env514.R (GTCTCGTGGGCTCGGAGATG TGTATAAGAGACAGGGGTTGCCAGTAGGCGGTTCC) according to previous studies (Chappell et al., 2017; Quigley et al., 2018, 2019). Briefly, HotStarTaq Plus Master mix (Qiagen), 0.2 µm env22.F, 0.2 µm env514R and 1 µl of sample DNA were combined for amplification at 95°C for 15 min initial denaturing, 40 cycles of 95°C denaturing for 30 s, 55°C annealing for 30 s and 72°C extension for 30 s, with a 5‐min final extension at 72°C. Amplicons were sequenced (MiSeq, Illumina) using the V3 300‐bp paired‐end chemistry after barcoding (eight rounds of tag‐addition amplification) at Ramaciotti Centre for Genomics.

### 2.4 KoRV operational taxonomic unit generation

KoRV sequence reads were filtered for length and primer‐trimmed with cutadapt (Martin, 2011), before forward and reverse amplicon reads were merged with flash (Magoc & Salzberg, 2011). Operational taxonomic units (OTUs) were generated with the qiime 1.9.1 software package (Caporaso et al., 2010) using the scripts pick_otus.py (set to 97% sequence identity, which uses the uclust algorithm to divide sequences into clusters), pick_rep_set.py (to retrieve a representative sequence from the created clusters) and map_reads_to_reference.py (set to 98% minimum percentage identify of match over at least 95% of both reference and read, which uses the usearch algorithm to map flash‐merged reads to the uclust‐generated reference sequences; Edgar, 2010). This process generated a KoRV OTU frequency table that listed the number of sequence reads from each koala sample that mapped to each KoRV OTU identified (Table S1). OTUs that contained fewer than 100 reads (across all samples) were removed from the data set and the remaining OTUs were blast searched (Altschul et al., 1990) against a library of known KoRV *env* sequences (generated from Chappell et al., 2017; Quigley et al., 2018) for initial identification. KoRV subtype was assigned based on the protein sequences of the RBD of each OTU. An OTU was defined as defective if the *env* gene sequence contained an in‐frame stop codon that would lead to premature truncation of the translated Env protein. KoRV OTUs generated in this study are available in GenBank under accession nos MN931399–MN931590.

### 2.5 KoRV subtype‐specific proviral copy number per cell quantification

Quantitative polymerase chain reaction (qPCR) was used to determine the koala β‐actin gene copy number (Shojima et al., 2013) and KoRV proviral *env* gene copy number from subtypes A, B, D and F (Quigley et al., 2019) in KoRV‐positive samples with sufficient material for analysis (*n* = 111). Briefly, the koala β‐actin gene was quantified using iTaq Universal SYBR green supermix (Bio‐Rad), 0.6 µm B‐actin‐K‐F (GAGACCTTCAACACCCCAGC), 0.6 µm B‐actin‐K‐R (GTGGGTCACACCATCACCAG) with 1 µl sample DNA using the programme: initial denaturing of 95°C for 5 min, followed by 40 cycles of denaturation at 94°C for 15 s, annealing at 60°C for 30 s, extension and data acquisition at 72°C for 30 s, followed by melt curve analysis from 65 to 95°C in 0.5°C increments. Subtype‐specific KoRV proviral *env* quantification was also carried out with iTaq Universal SYBR green supermix, a universal forward primer (KoRV_UF, TCYTGGGAACTGGRAAAAGAC) combined with subtype‐specific reverse primers (KoRV‐A_R, GGGTTCCCCAAGTGATCTG; KoRV‐B_R, GACTAACCCCCTGCCKACCT; KoRV‐D_R, GRTTCCCCAAGGKCGR; or KoRV‐F_R, GAYGTAAARCCAGGCCAAGG), with KoRV‐A primers used at a concentration of 0.3 µm while KoRV‐B, KoRV‐D and KoRV‐F primers were used at a concentration of 0.9 µm. Reactions were performed with an initial denaturing of 95°C for 5 min, followed by 40 cycles of denaturation at 94°C for 15 s, annealing at 54°C for 15 s, extension at 72°C for 15 s and data acquisition at 82°C for 10 s, followed by melt curve analysis from 65 to 95°C in 0.5°C increments. All reactions were run on a CFX 96 Touch System (Bio‐Rad) in duplicate and quantification was made by comparison to a standard curve generated from a dilution series of PCR products of a known concentration (10^7^ to 10^1^). Results were processed using bio‐rad cfx manager software. KoRV proviral copy number per cell was calculated by dividing the KoRV subtype‐specific proviral *env* gene copy number by the koala β‐actin copy number for each sample.

### 2.6 KoRV diversity statistics

Alpha diversity measures (Good's coverage, observed OTU and Simpson's Diversity Index [1‐D]) were calculated from the KoRV OTU frequency table with qiime 1.9.1 alpha_diversity.py. Statistical comparison of alpha diversity measures was performed using one‐way analysis of variance (ANOVA) with Tukey correction in spss software (IBM SPSS Statistics for Windows, version 24.0, IBM Corp.).

### 2.7 Maximum likelihood phylogeny

KoRV *env* RBD sequences (*n* = 192) were aligned using muscle, alongside GALV sequence (NC_001885.3) as an outgroup, and a maximum likelihood (ML) tree produced in the r package phangorn (Schliep, 2010). Using phangorn's modelTest function, the Kimura model with invariant sites and gamma distributed rate heterogeneity (K80 + Γ + I) was identified as the most appropriate for the KoRV sequence data. The K80 + Γ + I model was therefore used to produce a ML tree with 1000 bootstrap replicates. Clades were then annotated and the proportion of individuals from each geographical region for which a particular OTU was present were plotted alongside the tree using the ggtree package (Yu et al., 2017).

To visualize potential variations in the presence of different clades between geographical regions, OTU presence data were summarized by clade and geographical region to obtain the percentage of individuals in each location with OTUs from a particular clade corrected by the total number of OTUs within each clade.

### 2.8 Recombination detection

KoRV *env* sequences were checked for evidence of recombination signals using rdp4 4.100 (Martin et al., 2015). Sequences were aligned using muscle, followed by further manual editing in geneious prime 2020.1.1 (https://www.geneious.com). Sequences that differed from another by one or two bases were excluded from the analysis. Gaps were introduced within highly variable regions of the alignment in order to stagger poorly aligned sections of sequence and decrease the chance of erroneously identifying recombinants (Martin et al., 2017). Recombinants were accepted where they were detected by four or more of the methods in rdp4. Two sequence alignments were then exported: (a) excluding sequences with recombinant signals (*n* = 111 sequences remaining) and (b) with the recombinant regions of each sequence removed (*n* = 192 sequences but with suspected recombinant sections removed). These two alignments were then used to produce phylogenetic trees that were compared to the original phylogenetic tree using the complete sequences of all 192 OTUs detected. All three sets of data produced phylogenetic trees containing largely congruent clades. As such, the final phylogenetic trees presented represent the original complete sequence for each of the KoRV OTUs (*n* = 192).

### 2.9 Bayesian and neighbour joining phylogeny

For Bayesian analysis, KoRV proviral OTU sequences (*n* = 192) were aligned in mega‐x (Kumar et al., 2018) using muscle (Edgar, 2004) alongside GALV sequence (NC_001885.3) as an outgroup. A phylogenetic tree was produced in mrbayes (Ronquist & Huelsenbeck, 2003) using the GTR substitution model. The chain length was set to 6 × 10^6^, including a burn‐in of 6 × 10^5^, with sampling at every 500 iterations. Convergence was assessed using tracer 1.7.1 (Rambaut et al., 2018). The consensus Bayesian tree was imported into R to allow OTU data to be plotted using phyloseq (McMurdie & Holmes, 2013). A neighbour joining tree (*n* = 192) was also produced using the r packages phangorn (Schliep et al., 2017) and ape (Paradis & Schliep, 2019) using 10,000 and 1000 bootstrap replicates, respectively. The neighbour joining tree was also plotted alongside OTU data using phyloseq.

### 2.10 Comparison of KoRV OTU profiles by koala sample type and geographical region

KoRV proviral OTUs were analysed using phyloseq (McMurdie & Holmes, 2013) in R (R Core Team, 2014) to (a) investigate differences in KoRV proviral diversity between sampling regions and (b) determine whether KoRV proviral diversity differs by sample type (blood, urogenital swab, scat) for paired samples. Raw read counts were normalized and ordination plots were generated using nonmetric multidimensional scaling (NMDS).

### 2.11 Relationship between major KoRV‐A OTUs

Statistical tests to determine differences in the proportion of the three major KoRV‐A OTUs (A3001, A3002 and A3003) by region were carried out using the Kruskal–Wallis test and Dunn's test to make pairwise comparisons using the r packages (R Core Team, 2014) pgirmess (Giraudoux, 2018) and rstatix (Kassambara, 2020). Relationships between A3001, A3002 and A3003 were investigated using linear regression in R. For each region, linear regression was carried out for all combinations of the three major OTUs, with the following independent and response variables, respectively: A3001–A3003, A3001–A3002 and A3002–A3003.

## 3 RESULTS

### 3.1 KoRV proviral profiles from different sample types were largely consistent

Various koala sample types were collected for this study. To ensure that KoRV proviral profiles could be directly compared between sample types, three koala sample types (blood, urogenital tract swab and scat) were collected from 10 SEQLD koalas. A KoRV proviral profile was generated from each sample type and sample types were compared. The average reads per koala for proviruses determined from swab (199,596 ± 9765 reads) and blood (191,155 ± 18,301 reads) samples was significantly greater compared to scat samples (122,144 ± 19,063 reads).

**Figure 1:**
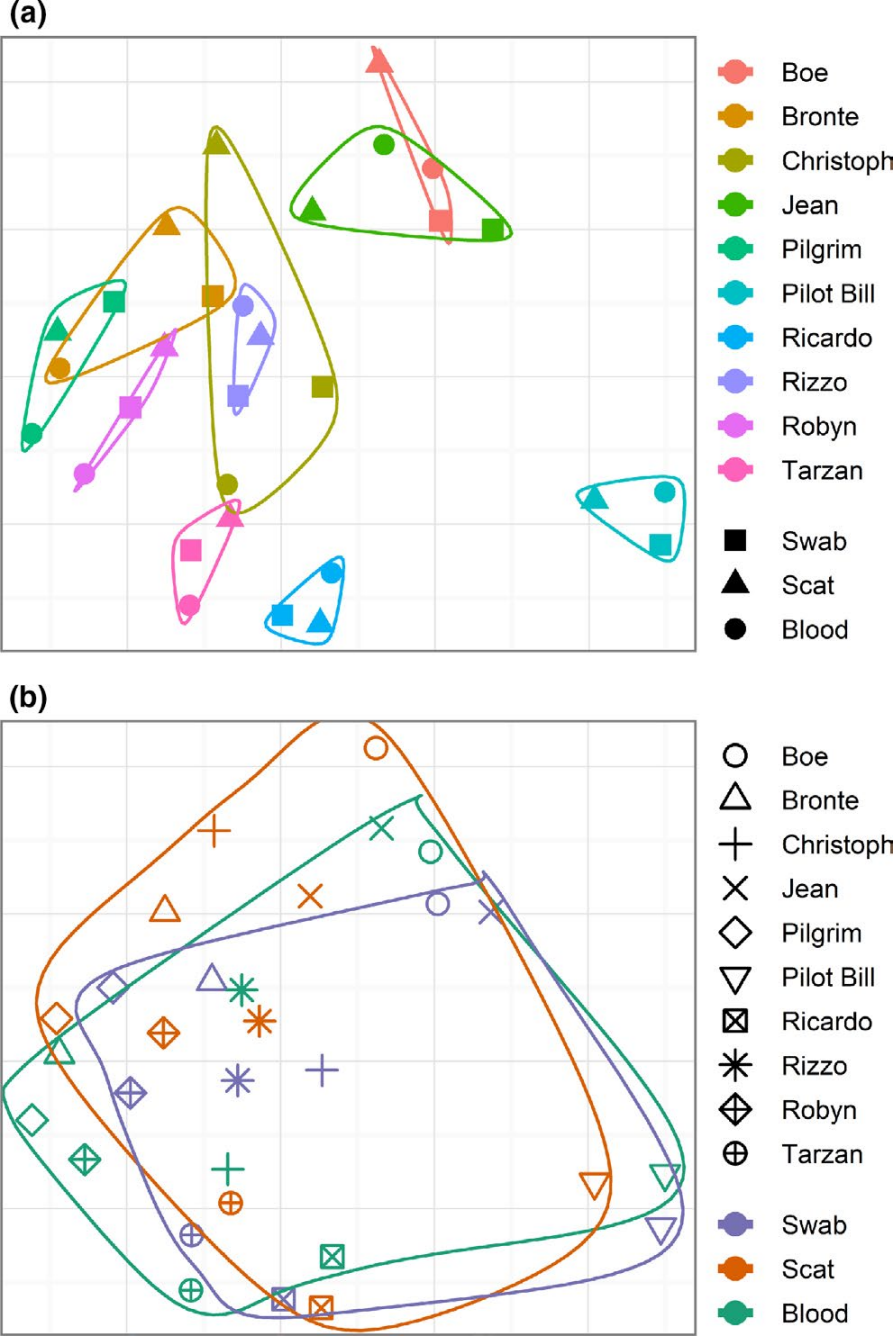
Non‐metric multidimensional scaling (NMDS) ordination using Bray–Curtis dissimilarity distance for KoRV provirus beta diversity of different sample types taken from the same koala. KoRV diversity profiles are coloured and grouped by koala (a) or sample type (b) for comparison

For each koala, a combined KoRV proviral profile from all three sample types was compared to each sample type individually, with 100%, 98% and 90% of the combined profile detected in the blood‐, swab‐ and scat‐only derived profiles, respectively (Table S2). OTUs not detected in the swab and scat samples were present at low copy number in the blood sample, indicating slight differences in proviral detection limits between sample types. However, ordination analysis found that proviral KoRV diversity clustered more significantly by individual koala than by sample type, indicating consistency in proviral profiles between sample types (Figure 1).

### 3.2 KoRV provirus detection was universal in koalas from QLD and NSW and less in VIC

**Figure 2:**
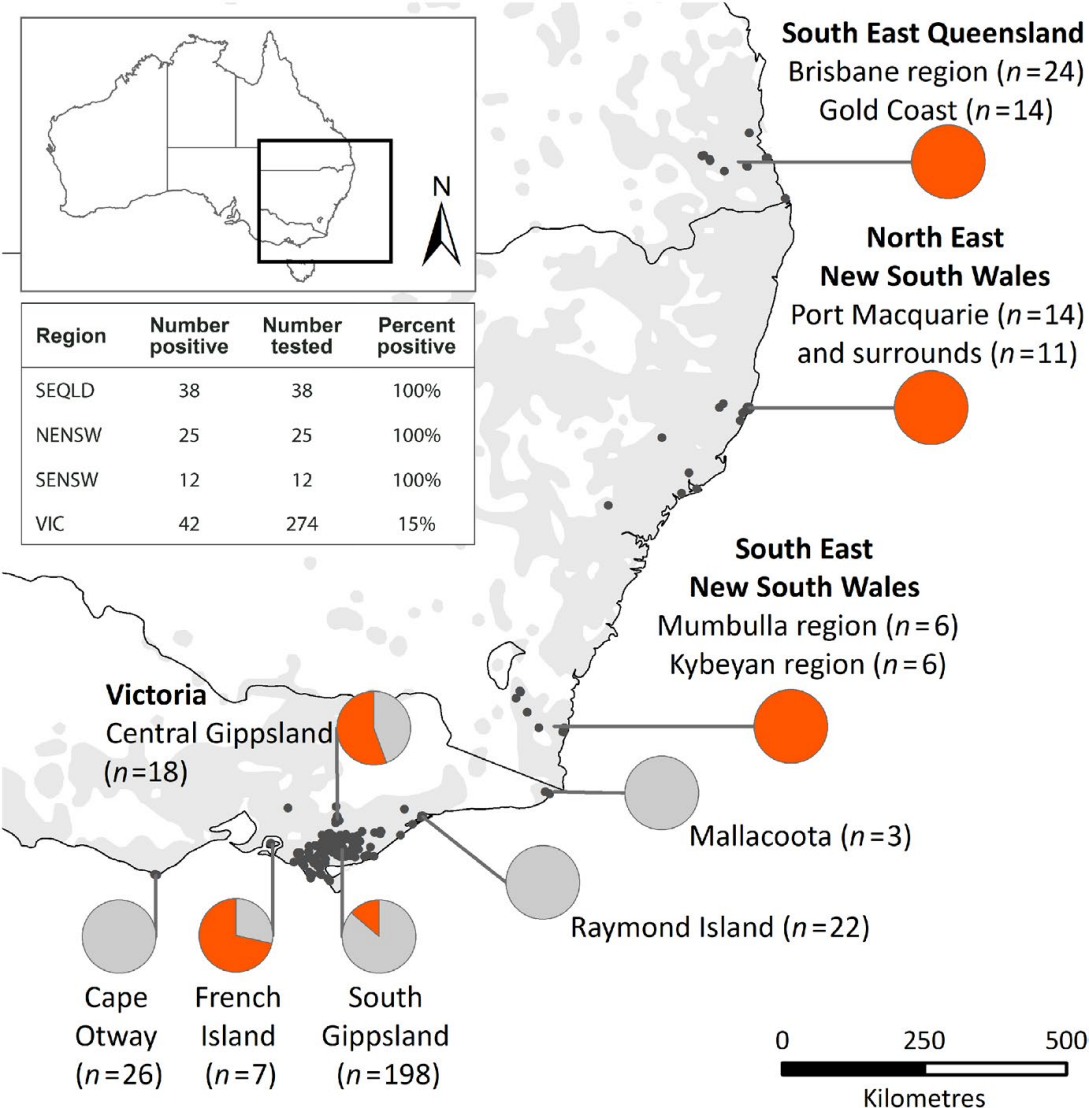
Summary of KoRV provirus detected by geographical region. Pie charts indicate the proportion of tested koalas in that region with detectable KoRV provirus *env* gene RBD. Dots indicate the location of the koala at sampling. Grey background shading on the map indicates the current koala distribution based on Australian Department of the Environment data. The inset table summarizes provirus detection rates by major region. SEQLD, southeast Queensland; NENSW, northeast New South Wales; SENSW, southeast New South Wales; VIC, Victoria

KoRV universal *env* primers env22.F and env514.R, which bind to conserved regions on either side of the RBD within the *env* gene, were used to test koala samples from across Australia. Of the 349 samples tested, 117 koalas were positive for KoRV provirus. By geographical region, 38/38 (100%) of SEQLD koalas, 25/25 (100%) of NENSW koalas, 12/12 (100%) of SENSW koalas and 42/274 (15%) of VIC koalas were KoRV provirus‐positive (Figure 2). The VIC koala sample set used in this study has been previously screened for KoRV‐A using a KoRV‐A‐specific qPCR assay (Wedrowicz et al., 2018). That report detected 33% (83/252) KoRV‐A positivity within the Victorian sample set (Wedrowicz et al., 2018). The difference in VIC KoRV detection rates between the previous study and this one is hypothesized to be due to the much larger assay amplicon in this study (490 bp) compared to the previously used screening assay amplicon (111 bp; Wedrowicz et al., 2018; Xu et al., 2013).

### 3.3 KoRV subtype‐specific provirus copies per cell

To measure the amount of KoRV provirus per cell by subtype, KoRV‐positive samples with enough material for analysis (*n* = 111) were quantified for copies of the koala β‐actin gene and KoRV proviral *env* gene specific to subtypes A, B, D and F. Koala β‐actin levels (representing the number of cells µl^−1^ of sample) ranged from 3.0 × 10^1^ to 4.7 × 10^6^ copies µl^−1^, with a median level of 2.2 × 10^4^ copies µl^−1^.

**Figure 3:**
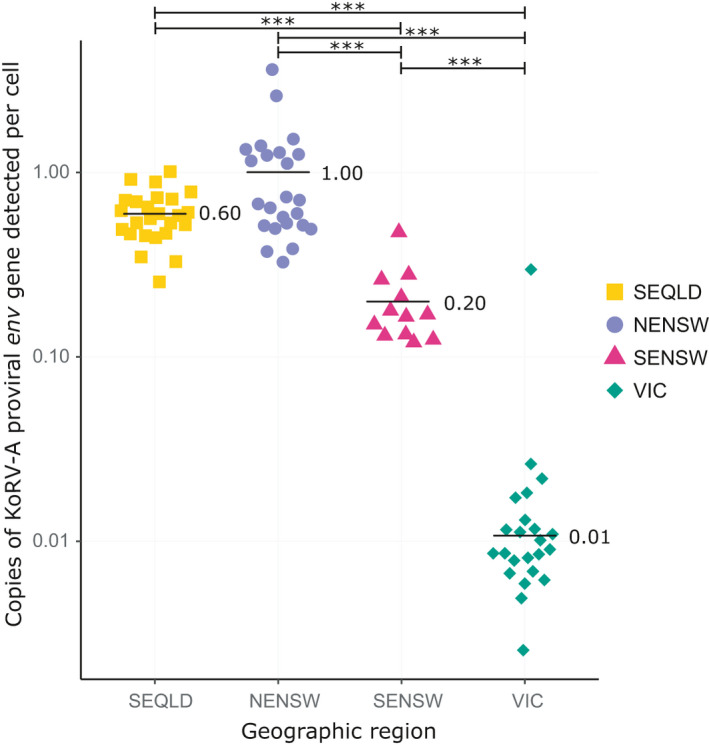
KoRV‐A proviral burden per cell by geographical region. Copies per cell were calculated by dividing the detected copies of KoRV‐A *env* gene provirus by the detected copies of the koala β‐actin gene. Mean copy number detected per group is indicated by the black bar. The VIC outlier sample was excluded from the mean and statistical calculations. Statistically significant differences between groups (*p* < .001 by pairwise comparisons using Wilcoxon rank sum exact test with the Benjamini & Hochberg (1995) *p* value adjustment to control the false discovery rate) are indicated by asterisks

Quantification of KoRV‐A proviral levels by geographical region determined the mean proviral copy number per cell to be 6.0 × 10^−1^ in SEQLD, 1.0 × 10^0^ in NENSW, 2.0 × 10^−1^ in SENSW and 1.0 × 10^−2^ in VIC (Figure 3). Interestingly, one VIC sample was an obivous outlier for this geographical region, with a KoRV‐A proviral copy number per cell of 3.0 × 10^−1^, which was much higher than the regional average (Figure 3). Examination of this sample's history revealed this koala was found after a bushfire and taken to a wildlife shelter south of its rescue location. Genetic analysis of this individual showed part of its ancestry was more closely related to NSW koalas than to VIC koalas (F. Wedrowicz *et al*., 2018), suggesting it could have been an unofficially translocated koala (or offspring thereof) or part of a low‐density remnant population in the Victorian highlands. Given its complex genetic geographical associations, this sample was included in Figure 3 but was not included in the calculation of the mean copy number per cell for VIC koalas.

Quantification of KoRV‐B, KoRV‐D and KoRV‐F provirual levels resulted in 90% of samples returning levels below detection (<1.0 × 10^1^ copies µl^−1^ of sample tested). From the 10% of samples with quantifiable provirus, the median copies per cell of KoRV‐B/D/F provirus was 1.2 × 10^−1^.

### 3.4 KoRV provirus OTU generation

Deep sequencing of the KoRV provirus *env* gene RBD amplicon from 117 positive koalas generated 32,827,062 total reads. Of these, 29,370,353 reads (89%) were identified as KoRV, with a median of 95% of reads within a sample mapping to KoRV OTUs. While it was observed that scat samples occasionally generated higher percentages of non‐KoRV reads, the lowest Good's coverage achieved in this data set was 0.9860 and the average Good's coverage achieved was 0.9997. These values indicate that sequencing was predicted to have sampled an average of 99.97% of the KoRV diversity present in each sample.

**Figure 4:**
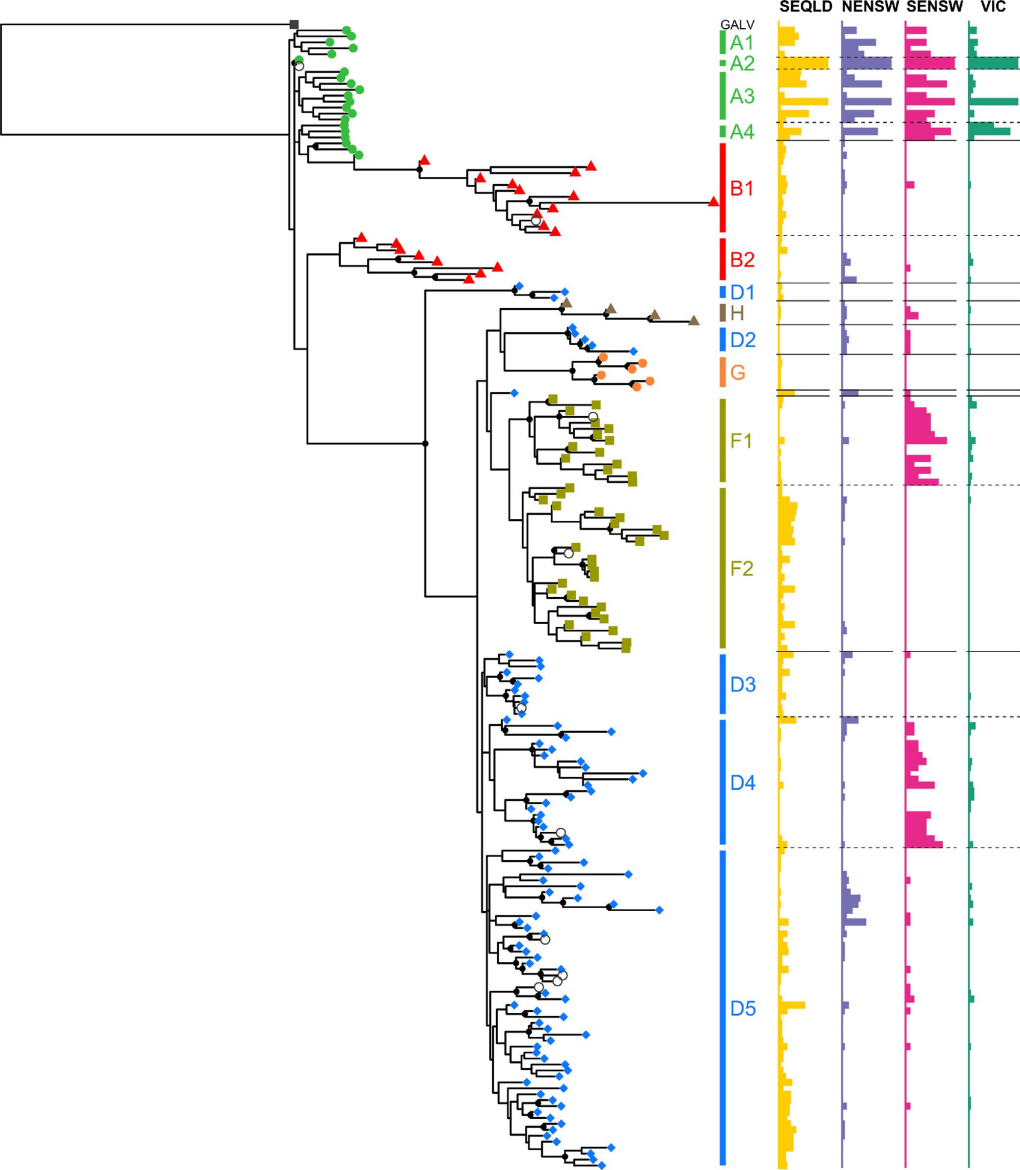
Maximum likelihood phylogenetic tree of the 192 OTUs detected, rooted to GALV (NC_001885.3). Tree tip symbols are coloured by subtype: KoRV‐A ((● green), KoRV‐B (▲ red), KoRV‐D (◆ blue), KoRV‐F (⬛ dark yellow), KoRV‐G (● orange), KoRV‐H (▲ brown), GALV (⬛ black) and defective KoRV OTUs (○ open circles). Black dots on a node represent >70% bootstrap confidence at that node. The horizontal bar plots to the right of the tree show the percentage of individuals within each population (SEQLD: yellow, NENSW: purple, SENSW: pink, VIC: green) for which each OTU was present. Horizontal lines across bar plots separate subtypes (solid lines) and clades (dotted lines)

The complete pan‐Australian data set generated 192 unique KoRV OTUs (Table 1; Figures 4 and S1a). Each OTU represents a unique KoRV variant, with each koala possessing an average of 16 different KoRV OTUs (ranging from three to 43 KoRV OTUs per koala). Using the amino acid sequence of the RBD, OTUs segregated into 22 KoRV‐A (21 intact, one defective), 21 KoRV‐B (20 intact, one defective), 96 KoRV‐D (90 intact, six defective), 43 KoRV‐F (41 intact, two defective), six KoRV‐G (all intact) and four KoRV‐H (all intact) subgroups. No KoRV‐C, ‐E or ‐I OTUs were detected in this data set.

**Table 1:** KoRV *env* OTU counts by subtype and geographical region

Subtype	Australia	SEQLD	NENSW	SENSW	VIC
A	21	19	18	14	14
B	20	16	9	2	4
D	90	64	37	30	21
F	41	35	9	12	10
G	6	6	0	0	0
H	4	2	4	2	1
Defective	10	7	1	4	1
Total	192	149	78	64	51

### 3.5 KoRV proviral diversity differed across Australia

The distribution of KoRV OTUs was not uniform across Australia (Table 1). SEQLD koalas contained the most diversity (149 OTUs), followed by NENSW koalas (78 OTUs), SENSW koalas (64 OTUs) and VIC koalas (51 OTUs; Table 1). Comparison of the number of OTUs detected between scat and blood samples in SEQLD (21 vs. 23 OTUs, respectively) as well as scat and ear clips from VIC (seven vs. eight OTUs, respectively) showed no significant difference, indicating that differences between regions were not likely to be impacted by sample type. Alpha diversity metrics reinforced these KoRV differences between geographical regions. The number of KoRV OTUs per koala varied from an average of 22 in SEQLD and 23 in SENSW to 13 in NENSW and seven in VIC. Simpson's Diversity Index (1‐D) also showed a significant trend for northern koalas (SEQLD and NENSW) to possess more diverse KoRV OTU profiles than southern koalas (Figures 2c and S1b).

### 3.6 Phylogeography differed between KoRV‐A and the other KoRV subtypes

To investigate the evolutionary relatedness of KoRV OTUs detected across Australia, a ML phylogenetic analysis of all KoRV proviral *env* OTUs was performed (Figure 4). The ML phylogeny segregated the OTUs into the expected three major KoRV clades (KoRV‐A, KoRV‐B and KoRV‐D/F/other), although strong bootstrap support was not achieved for deeper branches (Figure S2). Therefore, a neighbour‐joining phylogeny and Bayesian phylogeny were also generated to further investigate the phylogenetic relationships of KoRV *env* sequences (Figures S3 and S4). Both alternative phylogenetic approaches generated trees with very comparable clade structures to the ML approach, also without strong posterior probability or bootstrap support for deep branch points. Principal component analysis was used as an alternative methodological approach to confirm the OTU composition of each subtype, which supported the major KoRV subtype divisions detected in the phylogenies (Figure S5). Taken together, these multiple approaches supported the ML phylogeny as representing the relationships between KoRV *env* proviral OTUs.

**Figure 5:**
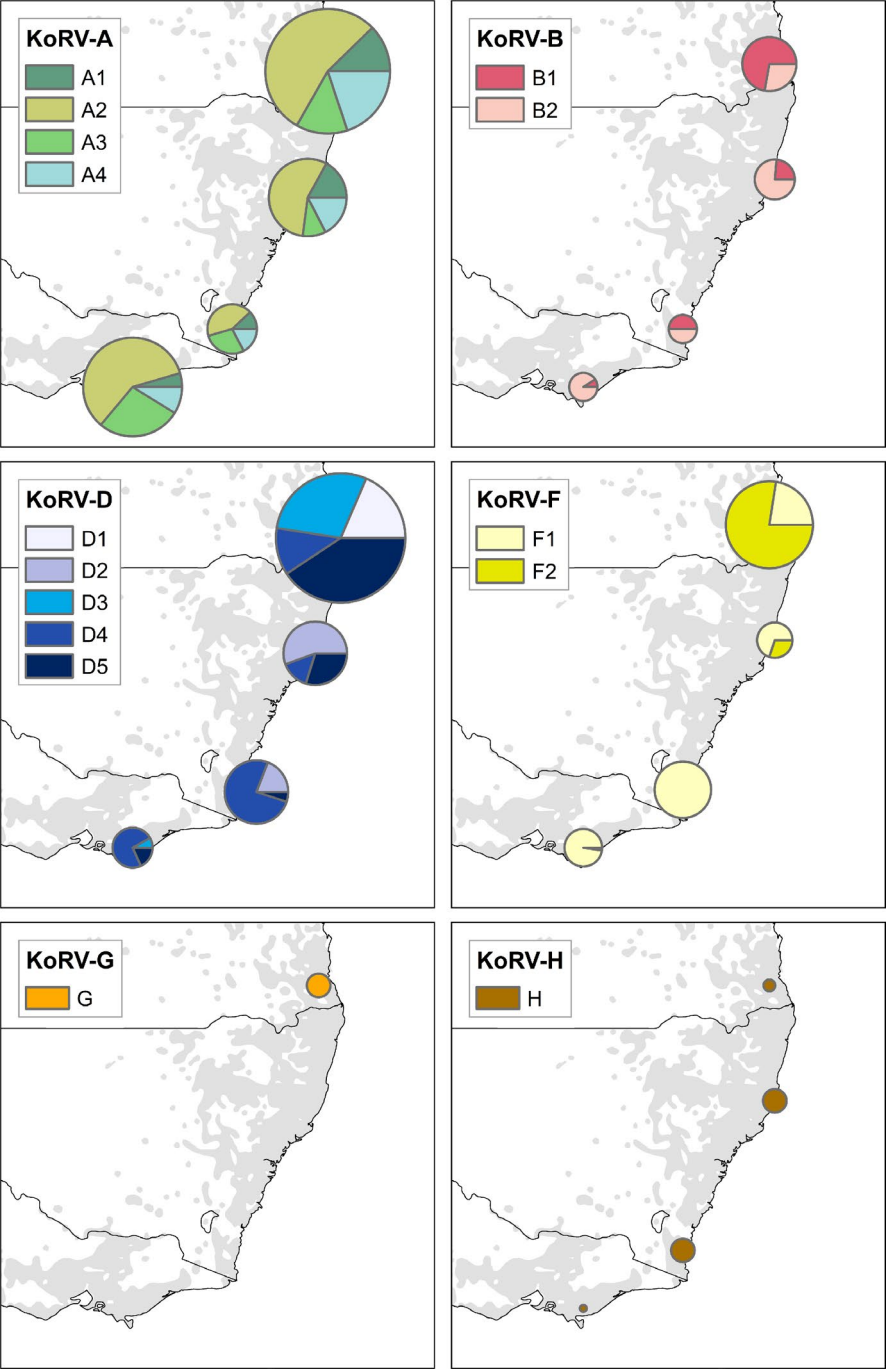
Pie charts showing the proportion of times that subtype clade OTUs were detected in individuals (where those OTUs were present). Pie chart size approximately represents the number of individuals within each population for which OTUs from each clade were detected

The KoRV *env* proviral ML phylogeny divided KoRV into clades with distinct geographical distributions. KoRV‐A comprised four clades with OTUs evenly distributed across Australia and representing koalas from all regions of Australia (Figures 4 and 5). KoRV‐B separated into two distinct clades, with clade B1 having a closer relationship to KoRV‐A and a higher prevalence in SEQLD while clade B2 branched further from KoRV‐A and had a higher prevalence in NENSW and VIC (Figures 4 and 5). KoRV‐D had the greatest diversity at five clades, with clades D1 and D3 having a higher prevalence in SEQLD, clade D5 having a higher prevalence in SEQLD and NENSW, clade D2 having a higher prevalence in NSW (both NENSW and SENSW), and clade D4 having a higher prevalence in SENSW and VIC (Figures 4 and 5). KoRV‐F appeared as two distinct clades within the KoRV‐D/F/other group, with clade F1 having a higher prevalence in NENSW, SENSW and VIC while clade F2 had a higher prevalence in SEQLD (Figures 4 and 5). Finally, KoRV‐G and KoRV‐H each branched into single clades within the KoRV‐D/F/other group, with KoRV‐G only detected in SEQLD and KoRV‐H detected at low levels across Australia (Figures 4 and 5).

### 3.7 Major KoRV‐A OTUs represented different proportions of a koala's KoRV provirus profile in different geographical regions across Australia

Of the 22 KoRV‐A OTUs identified, three (A3001, A3002 and A3003) were detected at significant levels in every KoRV‐positive koala tested (Figure S2). These KoRV‐A OTUs were present in all samples with detectable KoRV and comprised the majority of sampled reads. A3001 is identical to the originally published Hanger et al. (2000) KoRV sequence (AF151794.2), contains an attenuated CETAG Env protein motif (Oliveira et al., 2007) and is believed to represent the endogenous variant of KoRV‐A found in northern koalas. A3002 is a defective variant of A3001, where a 2‐bp insertion (at positions 218–219 of the amplicon) has created a frameshift leading to a stop codon in the middle of the RBD sequence. A3003 is a KoRV‐A variant with 15 single nucleotide polymorphisms (SNPs) compared to A3001 (leading to five nonsynonymous amino acid changes in the Env protein, including a return to the more virulent CETTG Env motif; Oliveira et al., 2007). Together, OTUs A3001, A3002 and A3003 accounted for an average of 97.0% reads from SEQLD samples, 99.2% reads from NENSW samples, 91.6% reads from SENSW samples and 99.6% reads from VIC samples.

**Figure 6:**
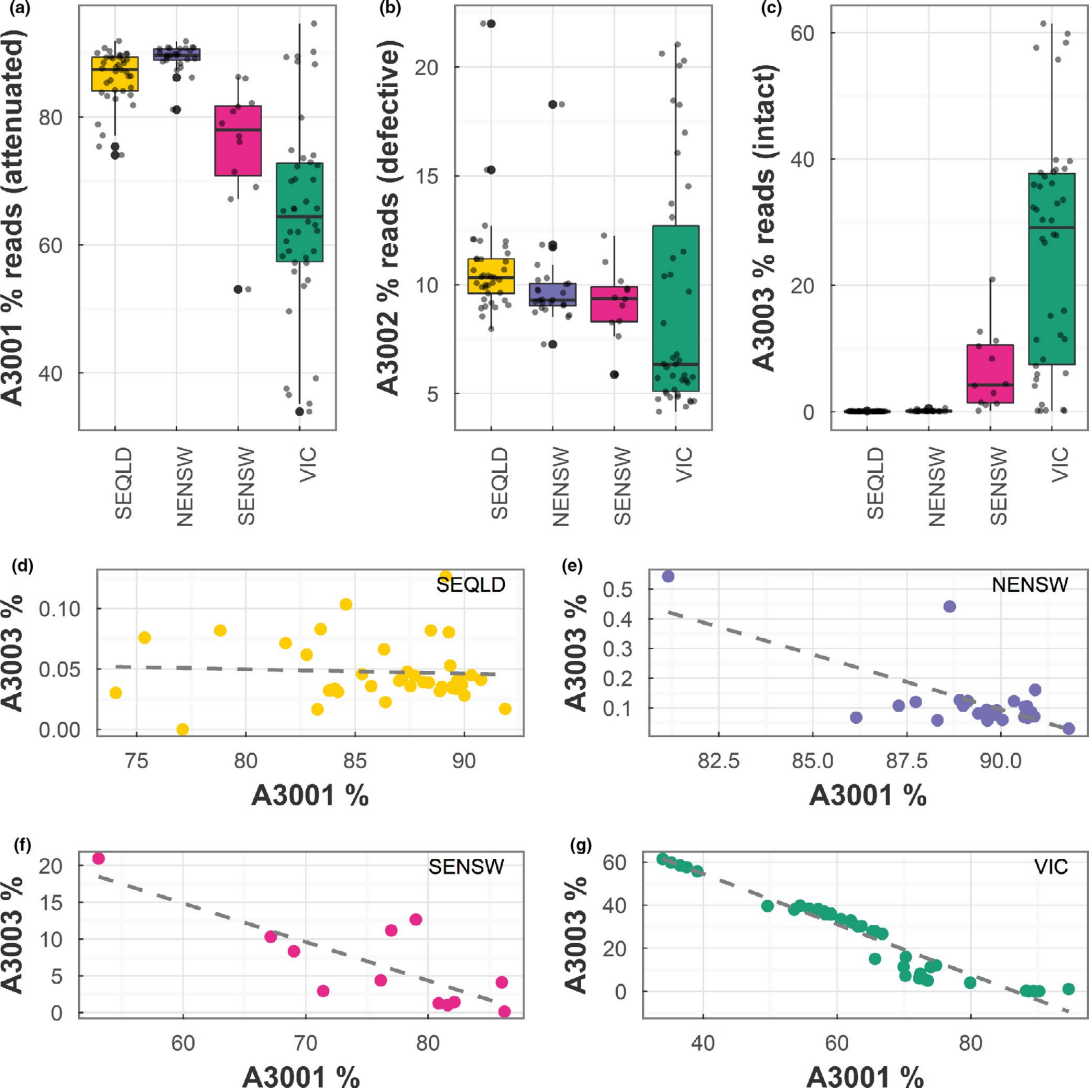
Percentage reads and correlation of the three universal KoRV‐A OTUs (A3001, A3002 and A3003) by region. Boxplots of average percentage abundance, overlaid with individual values (smaller grey points) for (a) A3001 (identical to the Hanger et al., 2000 KoRV sequence [endogenous variant in northern koalas], with an Env protein containing the attenuated CETAG motif), (b) A3002 (containing a frameshift leading to a truncated, defective envelope) and (c) A3003 (KoRV‐A variant with an Env protein containing the more virulent CETTG motif) detected in koalas by region. Correlation between the abundance of A3001 and A3003 detected in each koala in (d) southeast Queensland (SEQLD), (e) northeast New South Wales (NENSW), (s) southeast New South Wales (SENSW) and (g) Victoria (VIC). Regression axis labels indicate the percentage of reads attributed to each OTU. Regression equations were (d) SEQLD: *A3003* = –0.00035 *A3001* + 0.00078 (*R* ^2^ = 0.36%, *p* = .721), (e) NENSW: *A3003* = –0.037 *A3001* + 0.034 (*R* ^2^ = 46%, *p* = .0002), (f) SENSW: *A3003* = –0.52 *A3001* + 0.46 (*R* ^2^ = 62%, *p* = .002) and (g) VIC: *A3003* = –1.2 *A3001* + 1.01 (*R* ^2^ = 92%, *p* < 2.2 × 10^−16^)

The average percentage abundance of reads per koala for the three major KoRV‐A OTUs showed completely different patterns across the geographical regions studied (Figure 6a–c). For A3002 (the defective variant), the average percentage abundance of reads per koala was largely similar across the country ($\bar{x}$ = 9.9% reads, Figure 6b). A3002 proportions were not significantly different between regions except for between SEQLD and VIC koalas where A3002 was on average 1.5% greater in SEQLD (*p* = .002). Conversely, the average percentage abundance of reads per koala for A3001 (the suspected northern endogenous variant) decreased from north to south (Figure 6a) while the average percentage abundance of reads per koala from A3003 (a suspected exogenous variant) increased from north to south (Figure 6c). Statistical comparison of A3001 levels between regions showed that A3001 proportions were not significantly different between SEQLD–NENSW or SENSW–VIC but were between NENSW (89%) and SENSW (76%, *p* = .0001). On average, the proportion of A3001 in northern koalas (SEQLD/NENSW) was 20% greater than in southern koalas (SENSW/VIC), with A3001 decreasing from an average abundance of 87 ± 0.5% reads per koala in the north to 67 ± 2.0% reads per koala in the south (*p* < .005, Figure 6a). Conversely, A3003 increased dramatically from an average abundance of only 0.077 ± 0.01% reads per koala in the north to 21 ± 2.5% reads per koala in the south (*p* < .005, Figure 6c), differences in A3003 proportions were significant between all regions except for between SENSW and VIC.

Regression analyses between A3001, A3002 and A3003 KoRV‐A OTUs were undertaken to further investigate relationships between these major OTUs. There was no significant relationship detected between A3001 and A3003 reads per koala in SEQLD (*R* ^2^ = 0.036, *p* = .72, Figure 6d), but relationships were present in NENSW, SENSW and VIC (Figure 6e–g), with an increasing negative slope from north to south. Beginning in NENSW with a slope of –0.037 (*R* ^2^ = .46, *p* < .0005) and increasing to –0.52 (*R* ^2^ = .62, *p* = .002) in SENSW and –1.2 (*R* ^2^ = .92, *p* < .0005) in VIC, as A3003 reads represented more of the detected provirus per koala, A3001 represented less of the detected provirus per koala (Figure 6e–g). However, OTU A3001 was still the most abundant KoRV‐A proviral OTU sequence detected in the south ($\bar{x}$ = 67% of reads), on average at least three times more abundant than A3003 ($\bar{x}$ = 21% of reads). Interestingly, there were a mixture of relationships between either A3001 or A3003 and A3002 (the defective variant) across the geographical regions (Figures S6 and S7), but no biologically relevant patterns were detected.

## 4 DISCUSSION

This study set out to analyse KoRV *env* gene RBD diversity in koalas to address fundamental questions in a natural system undergoing current retroviral diversification and endogenization. By combining knowledge of each koala's location at sampling and the copy number of KoRV‐A provirus per cell, this study revealed that major KoRV‐A provirus variants have already spread across the koala's range but that the levels of provirus per cell and the dynamics of these variants differed across Australia. This study also highlights that KoRV‐B, KoRV‐D, KoRV‐F, KoRV‐G and KoRV‐H provirus variants appear to have undergone lineage diversification from KoRV‐A in geographically distinct regions. While this analysis could not date the timing of the KoRV‐A spread or the non‐KoRV‐A diversification, it does suggest that KoRV‐A has already geographically spread across Australia, and differences in total KoRV or KoRV‐A prevalence across the country are not due to a lack of opportunity for koalas to be exposed to this virus.

With a body of evidence indicating that only KoRV‐A appears to be currently undergoing endogenization (Cui et al., 2016; Ishida et al., 2015; Tsangaras et al., 2014; Xu & Eiden, 2015), this study set out to determine the KoRV proviral copy number per cell for individual subtypes of KoRV across Australia. The only assays able to distinguish KoRV subtypes target the RBD within the *env* gene (Quigley et al., 2019). As such, these assays were used in conjunction with an established koala β‐actin quantification assay (Shojima et al., 2013) to determine the proviral copy per cell of each subtype. Three general levels of KoRV‐A proviral copies per cell were detected across Australia: SEQLD and NENSW koalas had levels around one proviral copy per cell, SENSW koalas had levels around one proviral copy per five cells, and VIC koalas had levels of around one proviral copy per 100 cells. Of note, while the average SEQLD KoRV‐A proviral level of 6.0 × 10^−1^ copies per cell was lower than previous QLD reports for total KoRV proviral copies per cell, several known factors would be expected to contribute to a lower KoRV‐A‐specific value. Previous estimates of QLD proviral copies per cell have targeted the KoRV *pol* gene, which detects all KoRV subtypes (Sarker et al., 2020; Simmons et al., 2012). When KoRV provirus was examined at the subtype level in the koala genome, KoRV‐A was found to represent about half of the total KoRV provirus present in a QLD koala cell (Hobbs et al., 2017; Sarker et al., 2020; Simmons et al., 2012). Additionally, inferring the presence of various KoRV subtypes requires assaying the most variable region of the KoRV virus (the RBD within the *env* gene). SEQLD had the greatest diversity of KoRV‐A OTUs, defined by differences in the RBD. This diversity may have limited the quantification of all KoRV‐A variants present in the genome. With these factors taken into consideration, the levels of both SEQLD (6.0 × 10^−1^ proviral copies per cell) and NENSW (1.0 × 10^0^ proviral copies per cell) KoRV‐A are within a reasonable range for the detection of a possible endogenous provirus. By comparison, the detected KoRV‐A proviral levels in VIC koalas (1.0 × 10^−2^ copies per cell), as well as the detected proviral levels of KoRV‐B, KoRV‐D and KoRV‐F (≤1.2 × 10^−1^ copies per cell) fall within a reasonable range for the detection of an exogenous provirus. Detecting an average of 2.0 × 10^−1^ KoRV‐A proviral copies per cell from SENSW koalas is more challenging to interpret and merits further discussion.

In this study, the distribution and characteristics of the three major KoRV‐A variants detected in every KoRV‐positive koala created an opportunity to hypothesize about their endogenous/exogenous states. The KoRV‐A variant A3001 appears to be the best adapted KoRV variant in koalas, having by far the highest proviral levels in any koala tested. This suggests that its RBD is very well adapted to its cell receptor in koalas, the sodium‐dependent phosphate transporter 1 (PiT1; Oliveira et al., 2006). In addition, A3001 has an attenuated CETAG Env motif, known to reduce viral syncytia‐inducing capability (Oliveira et al., 2007), which could be advantageous for an endogenous retrovirus. With A3001 appearing to have begun endogenization in northern koalas, it could be expected that processes such as superinfection exclusion and piwi‐acting RNA would prevent other KoRV‐A variants, such as A3003, from establishing significant infection in the same koala population (Yu et al., 2019). Strong selection from A3001 via homologous interference may have driven adaptation in A3003 to remain viable (a situation that has been documented with escape of Jaagsiekte sheep retrovirus provirus; Arnaud et al., 2007). Also potentially aiding A3003’s survival is the fact that A3003 possesses the more virulent CETTG Env motif (Oliveira et al., 2007), which is expected to allow it greater infectivity between cells. Hence, in koalas from SEQLD and NENSW, where A3001 is believed to be endogenous, it is the major KoRV‐A variant. In this location, A3003 maintains a minimal background presence with no or minimal correlation to A3001 proviral levels. However, in koalas where A3001 is not predominantly endogenous, both A3001 and A3003 compete directly for koala cell infection exogenously. Differences between A3001 and A3003 allow for both variants to exist in the koala population to what appears to be a KoRV subtype carrying capacity. At this capacity, increases in the presence of one KoRV‐A variant results in decreases in the presence of the other variant. Hence, in koalas where KoRV‐A appears predominantly exogenous (VIC), A3001 and A3003 have a strong (0.92) negative correlation. Finally, in a geographical area where endogenous incorporation of KoRV‐A is just beginning, we would expect to see a mixture of the two extremes. A newly endogenous A3001 variant would still be dominant in koalas, although provirus levels per cell may be low. An exogenous A3003 variant may still have some correlation to A3001 if A3001 proviral levels have not saturated the KoRV carrying capacity in the population. Strikingly, this relationship describes the situation found in SENSW. This reasoning leads to the hypothesis and first experimental support to designate SENSW as the geographical area where endogenous KoRV‐A is becoming less prominent in the koala population.

Phylogeographical analysis revealed that KoRV‐A variants have a uniform presence across Australia. However, despite this national pattern, KoRV still has a higher prevalence and diversity in northern koalas compared to southern koalas. Several factors may be contributing to this continued north–south KoRV difference. One hypothesis is that KoRV was introduced into northern Australian koalas from a spill‐over event from bats or rodents (Greenwood et al., 2018; Xu & Eiden, 2015). If KoRV originally spread through the koala population from north to south, KoRV may have had more time to diversify in northern koala populations compared to southern koala populations. Another factor may be that KoRV endogenization in northern koalas has generated a much larger reservoir of KoRV to diversify and spread than exogenous‐only KoRV in southern koalas. With endogenization leading to at least one proviral copy per cell, koala populations with endogenous virus would carry a large KoRV proviral burden. It has been shown that KoRV is still being actively transcribed in northern koalas (Quigley et al., 2019; Sarker et al., 2019, 2020), creating a substantial pool of viral genetic material to mutate, diversify and spread. Given this environment, it may not be surprising that the northern geographical regions have 100% KoRV prevalence and substantial KoRV diversity. Conversely, this study and others have found that KoRV provirus levels in southern koalas are commonly much less than one proviral copy per cell (Simmons et al., 2012). This low proviral load creates a technical challenge for detecting KoRV and less opportunity for natural diversification in the population. Finally, recent investigations have suggested that KoRV may, in fact, actually be more prevalent in southern koala populations than first estimated. This appears to be due to high rates of defective KoRV variants in these southern animals, potentially rendering tests targeting certain regions of KoRV ineffective (Sarker et al., 2020). Together, these factors may start to explain the different diversity and apparent prevalence of KoRV across Australia, given the finding that KoRV appears to have already spread across the koala's natural range.

Distinct from the phylogeographical pattern of KoRV‐A, the other KoRV subtypes revealed distinct lineage diversification in different geographical regions across Australia. This pattern suggests that KoRV subtype diversity has emerged repeatedly within regionally separated areas, as opposed to have being spread uniformly throughout the national koala population. The fragmentation of koala populations across Australia, occurring over the past 200 years, has no doubt contributed to this regional separation of both animals and virus.

Interestingly, this is the first report of KoRV‐B sequences appearing paraphyletic, with clade B1 sequences branching within the KoRV‐A clade and clade B2 sequences branching separately. KoRV‐B clade B2 appears to represent a novel lineage diversification of KoRV‐B detected primarily from New South Wales and Victorian koalas (Figure 4). In the neighbour joining and Bayesian trees (Figures S3 and S4), the two KoRV‐B clades are distinguishable within a monophyletic KoRV‐B branch, suggesting that the distinction between KoRV‐B being monophyletic or paraphyletic depends on the methodology: the ML tree (Figures 3 and S2) and minimum spanning tree (Figure S1) identify distinct KoRV sequences as the most recent common ancestor for each KoRV‐B clade while the neighbour joining and Bayesian trees identify a common KoRV sequence as the most recent common ancestor to both KoRV‐B clades. This distinction is unlikely to have biological impact, as both KoRV‐B clades have similar receptor binding domain regions. Additionally, it has not been a requirement for KoRV subtypes to represent monophyletic sequences, as KoRV‐D has always been described as a paraphyletic subtype (Chappell et al., 2017; Sarker et al., 2019). This finding does highlight that different populations of koalas harbour novel diversifications of KoRV that will continue to challenge our understanding of KoRV evolution and how we attempt to group and classify strains.

Finally, as a technical note, this study found that multiple subtypes of KoRV provirus could be detected consistently from koala blood, urogenital swabs and scat. This indicates that provirus from different KoRV subtypes was not substantially different between peripheral blood mononuclear cells found in blood and epithelial cells and tissue resident immune cells that would be collected by urogenital swabbing or gastrointestinal tract sloughing into scat. This consistency of KoRV proviral profiles gave an experimentally verified base to combine KoRV proviral profiles from multiple sample types for analysis in this study. While the cell receptor(s) for the non‐KoRV‐A/B subtypes are still unknown, the KoRV‐A PiT1 receptor and KoRV‐B thiamine transporter‐1 (THTR1) receptor are known to have ubiquitous tissue expression (Ganapathy et al., 2004; Johann et al., 1992). As such, it was not surprising to detect comparable KoRV provirus profiles from different koala samples containing different cell types. Additionally, these findings suggest strongly that the receptors for the non‐KoRV‐A/B subtypes will also be universal transporters common to most, if not all, cell types.

KoRV has a complex and continually evolving relationship with koalas. This study found evidence for both endogenous and exogenous KoRV‐A variants in SEQLD and NENSW koalas, with a transition in SENSW koalas towards predominantly exogenous KoRV‐A in VIC koalas. Sequence and abundance differences between major KoRV‐A variants offered clues as to how viral variants, with possible differences in endogenization status, may coexist within an animal. This study also found that KoRV‐B to KoRV‐H have a different diversification pattern to KoRV‐A, indicating that there are distinct differences between KoRV‐A and the other recognized KoRV subtypes. Continuing to expand our understanding of KoRV and how it interacts with koalas will reveal important information about managing this virus in koalas specifically and will add general information about retroviral evolution and endogenization in a new host.

## AUTHOR CONTRIBUTIONS

B.L.Q. and F.W. designed the research, performed the research, analysed the data and wrote the paper. F.H. and P.T. designed the research, supervised the research and wrote the paper.

## Supporting information


Figures S1‐S7

Tables S1‐S2

## Data Availability

DNA sequences: KoRV OTU sequences generated in this study can be found at GenBank under accession nos MN931399–MN931590. Raw data files can be obtained from the authors on request.
