# Supplementary material for: Phylogenetic and geographical analysis of a retrovirus during the early stages of endogenous adaptation and exogenous spread in a new host
Source: Mol Ecol. 2020 Dec 30;30(11):2626–40. doi: 10.1111/mec.15735 (PMC8246579; doi:10.1111/mec.15735)
Supplement: Supplementary file 1 — Figures S1‐S7 [file MEC-30-2626-s001.pdf]

MOLECULAR ECOLOGY

Supplemental Information for:

Phylogenetic and geographical analysis of a retrovirus during the early stages of endogenous adaptation and exogenous spread in a new host

Bonnie L Quigley, Faye Wedrowicz, Fiona Hogan, Peter Timms

Table of Contents:

**Supplementary figure 1**<br>KoRV provirus *env* gene RBD region prevalence and diversity.	Page 2
**Supplementary figure 2**<br>Maximum likelihood phylogenetic tree (extended version of Figure 3) of KoRV OTUs detected across Australia.	Page 3-4
**Supplementary figure 3**<br>Neighbour joining phylogenetic tree of KoRV OTUs detected across Australia.	Page 5-6
**Supplementary figure 4**<br>Bayesian analysis-based phylogenetic tree of KoRV OTUs detected across Australia.	Page 7-8
**Supplementary figure 5**<br>Principal components analysis (PCA) applied directly to KoRV OTU sequence data.	Page 9
**Supplementary figure 6**<br>Correlation between the abundance of A3001 and A3002 detected in each koala	Page 10
**Supplementary figure 7**<br>Correlation between the abundance of A3002 and A3003 detected in each koala	Page 11

MOLECULAR ECOLOGY

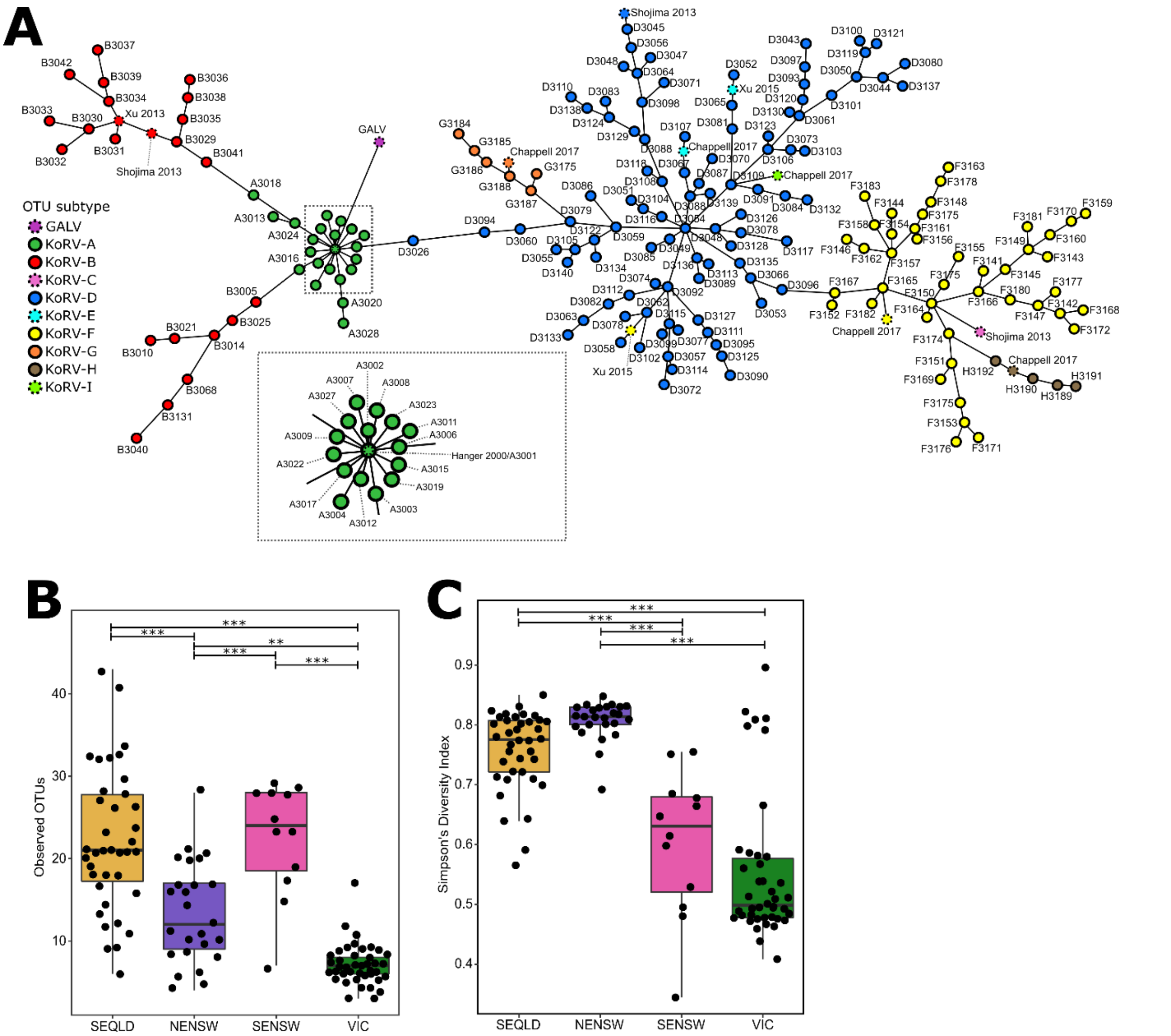

Supplementary Figure 1. KoRV provirus *env* gene RBD region prevalence and diversity. A minimum spanning tree were generated to diagram the relatedness of KoRV proviral OTUs (represented by circular nodes) (A). The tree were generated from aligned DNA OTU sequences using the PHYLOViZ Online site (http://online.phyloviz.net/index) with the programs default goeBURST algorithm. Reference sequences are indicated with a dashed outline and include KoRV-A (Hanger 2000 AF151794.2), KoRV-B (Shojima 2013 AB822554.1 and Xu 2013 KC779547.1), KoRV-C (Shojima 2013 AB828005.1), KoRV-D (Shojima 2013 AB828004.1), KoRV-E (Chappell 2017 (D15) KX588043.1 and Xu 2015 KU533853.1), KoRV-F (Chappell 2017 (F12) KX587994.1 and Xu 2015 KU533852.1), KoRV-G (Chappell 2017 (G3) KX587961.1), KoRV-H (Chappell 2017 (H1) KX588036.1), KoRV-I (Chappell 2017 (I1) KX588021.1), and gibbon ape leukemia virus (GALV) (Wilson U20589.1:465-1019). The alpha diversity measures of observed OTUs per sample (B) and Simpson's Diversity Index (C) are also presented by region with one-way ANOVA ($F(3,113) = 44.453$, $p < 0.001$ and $F(3,113) = 54.777$, $p < 0.001$, respectively).

MOLECULAR ECOLOGY

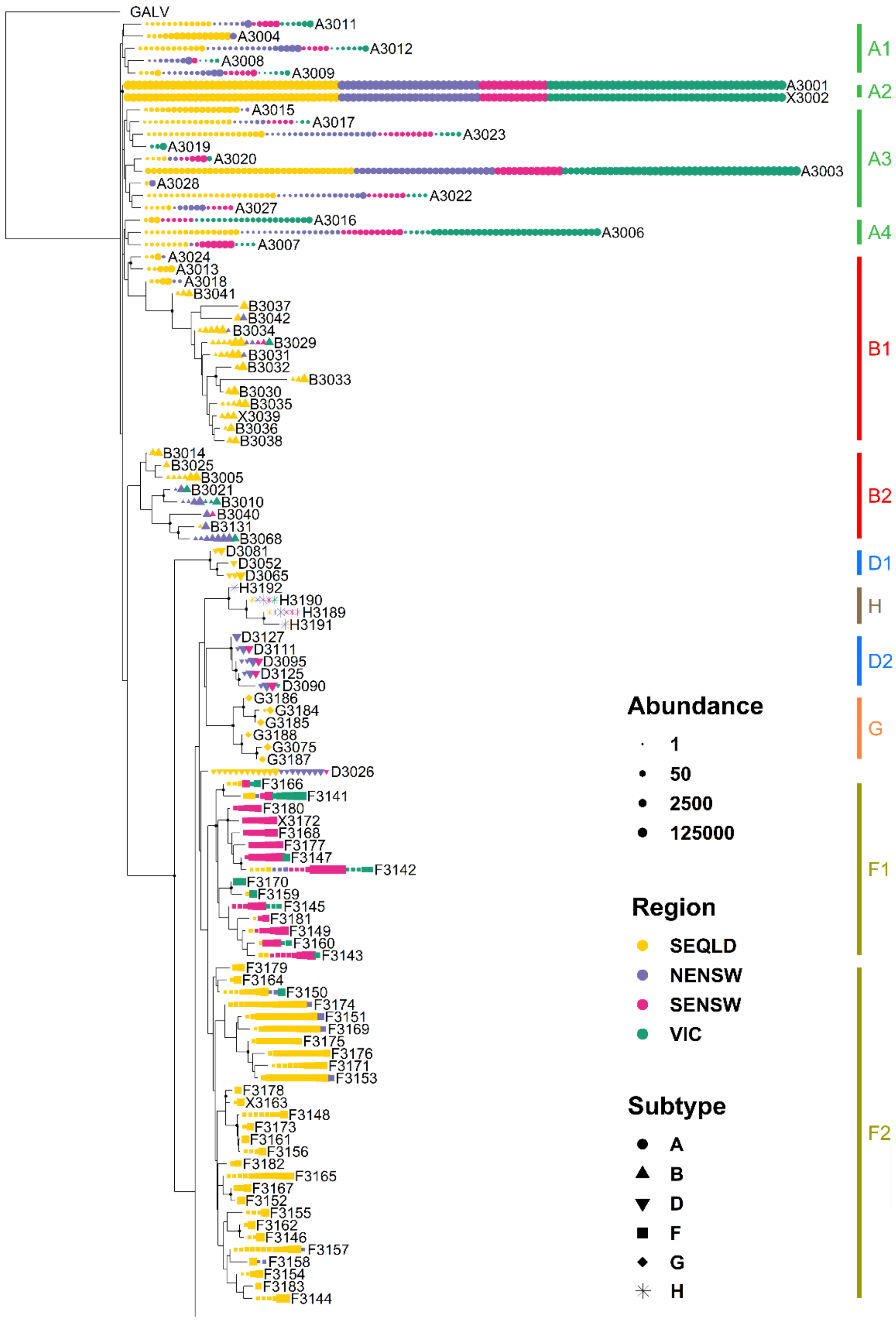

Supplementary Figure 2. Maximum likelihood phylogenetic tree (part 1)

MOLECULAR ECOLOGY

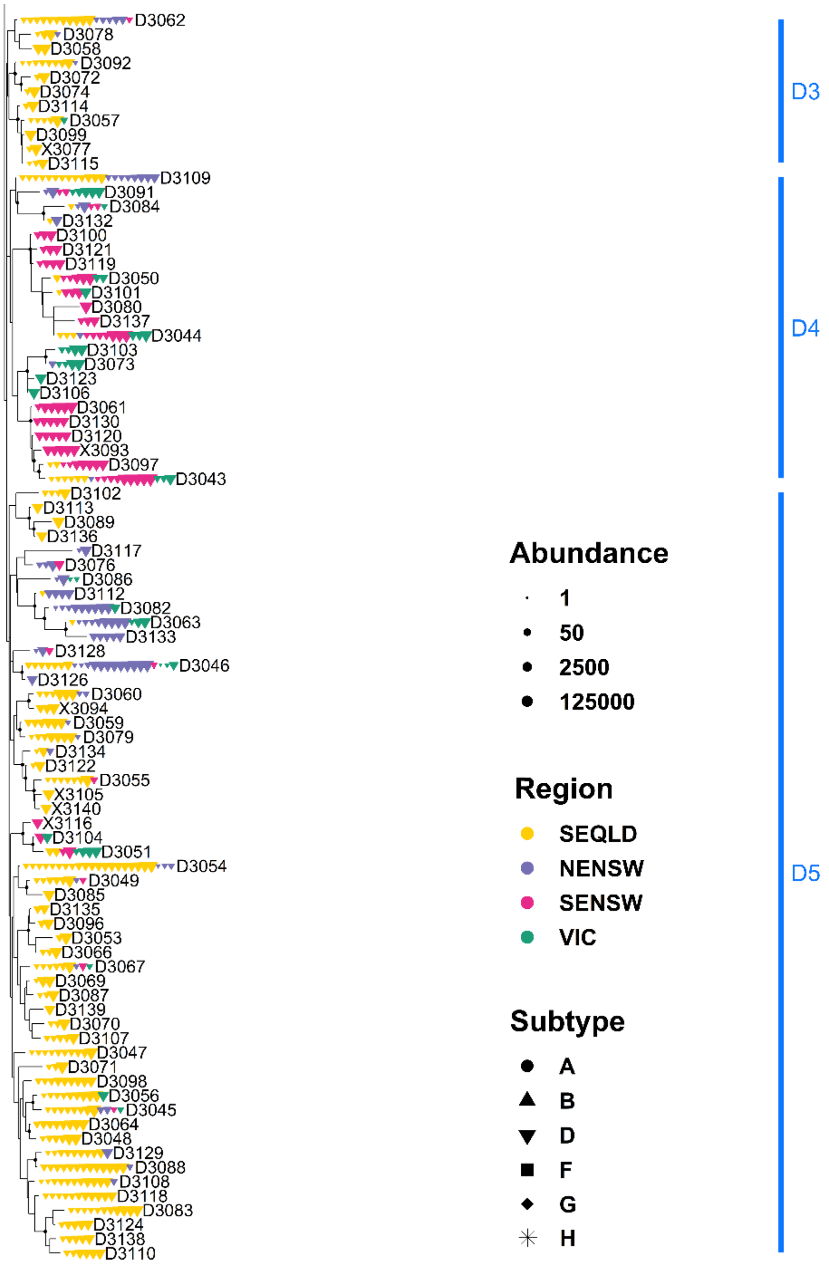

Supplementary Figure 2. Maximum likelihood phylogenetic tree (extended version of Figure 3) of KoRV OTUs detected across Australia. Each branch represents a detected KoRV OTU, with individual koalas possessing that OTU represented next to the branch by a symbol. Symbols indicate the subtype of the OTU (shape), the region that koala is from (colour) and the abundance of this OTU's sequence reads from that koala (size). Major clades are labelled at the right of the figure. DNA sequences were aligned using MUSCLE and included GALV sequence NC_001885.3 as the outgroup. Confidence in tree nodes was estimated using 1000 bootstrap replicates. Nodes with 70% or greater bootstrap support are marked with a black point. OTU labels starting with an X indicate defective *env* sequences.

MOLECULAR ECOLOGY

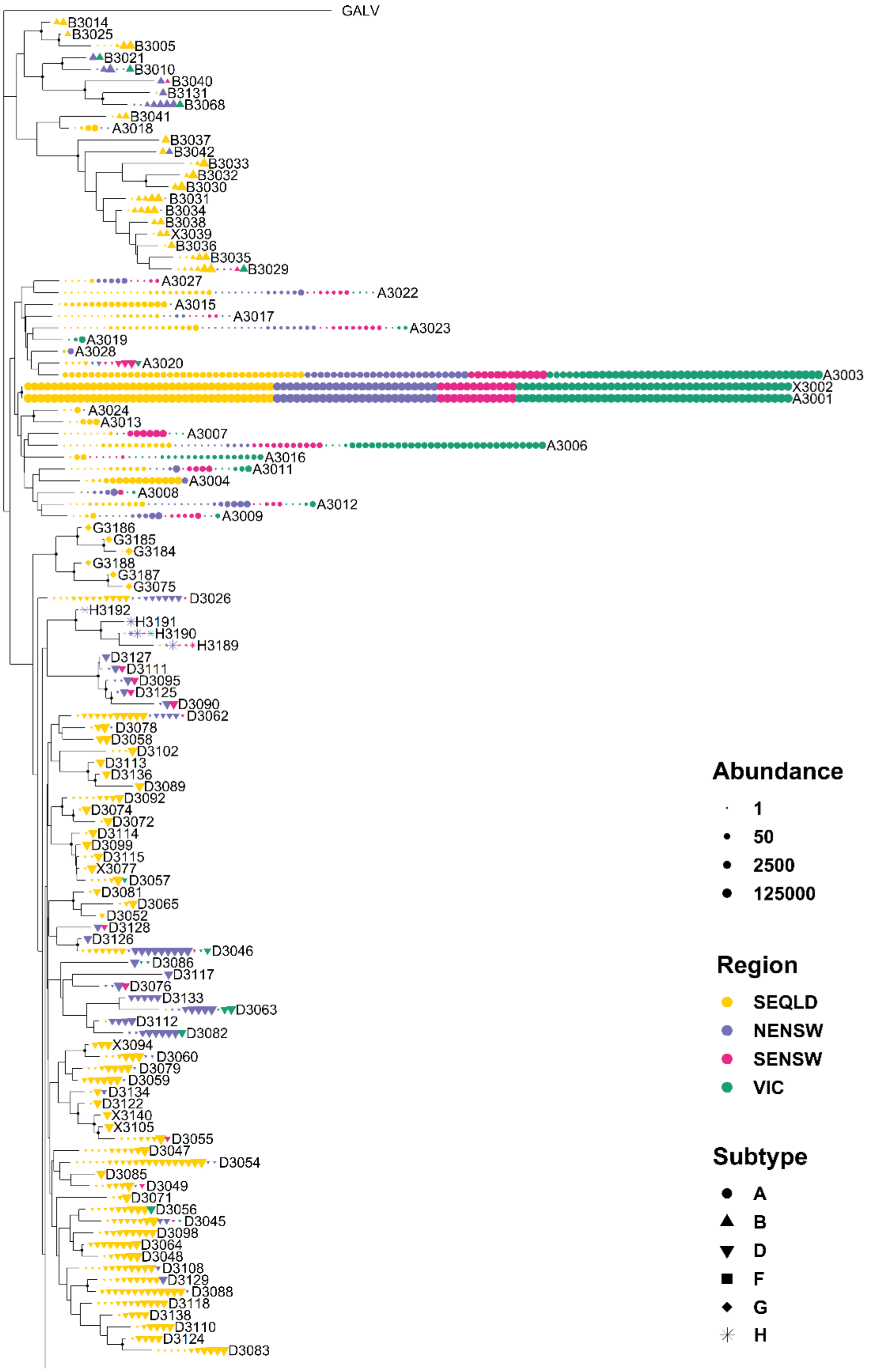

Supplementary Figure 3. Neighbour joining phylogenetic tree (part 1)

MOLECULAR ECOLOGY

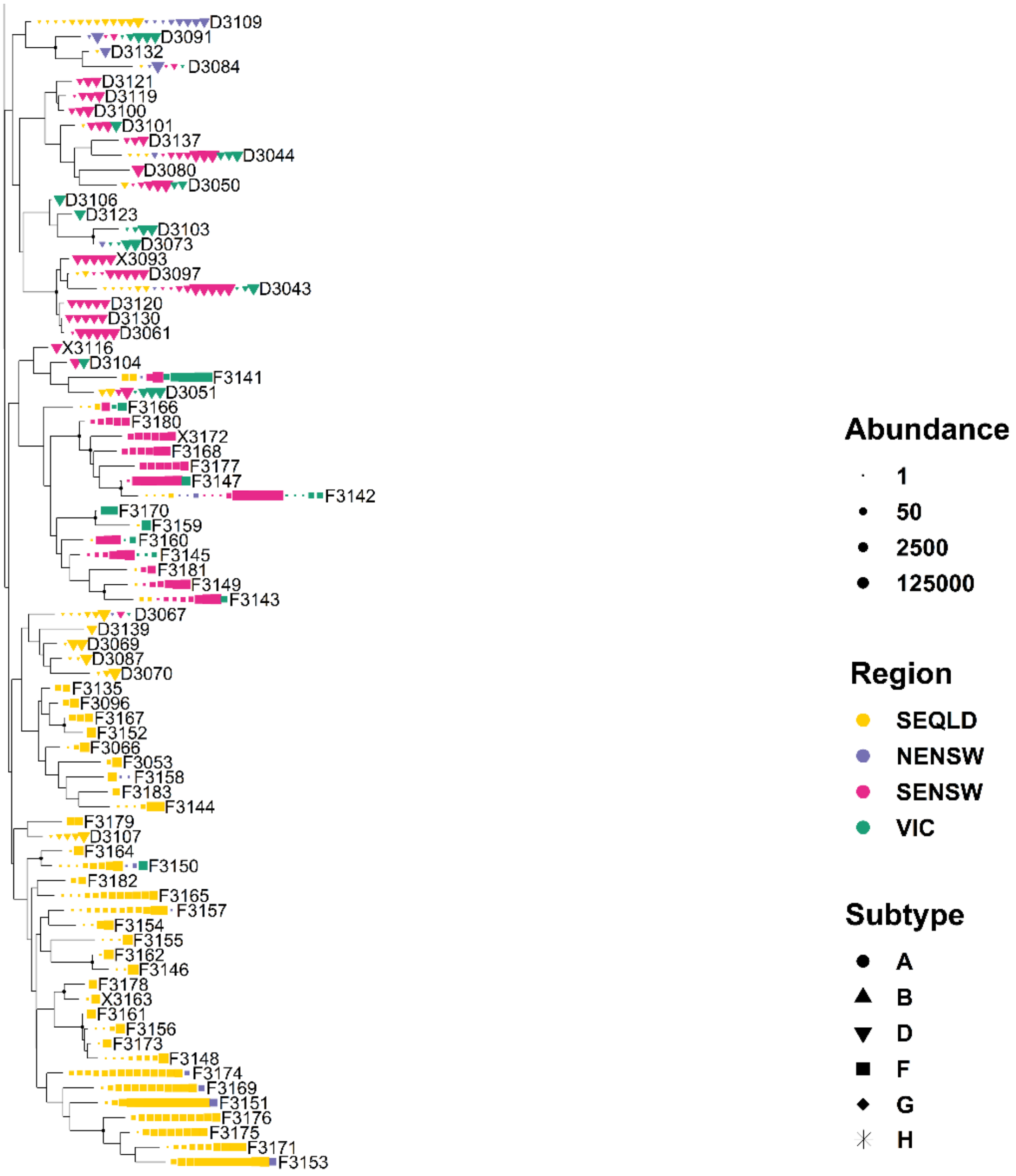

Supplementary Figure 3. Neighbour joining phylogenetic tree of KoRV OTUs detected across Australia. Each branch represents a detected KoRV OTU, with individual koalas possessing that OTU represented next to the branch by a symbol. Symbols indicate the subtype of the OTU (shape), the region that koala is from (colour) and the abundance of this OTU's sequence reads from that koala (size). The NJ tree was produced with the *phangorn* and *ape* packages using the Jukes-Cantor distance model. Confidence in tree nodes was estimated using 10,000 bootstrap replicates and nodes with 70% or greater bootstrap support are marked with a black point. OTU labels starting with an X indicate defective *env* sequences.

MOLECULAR ECOLOGY

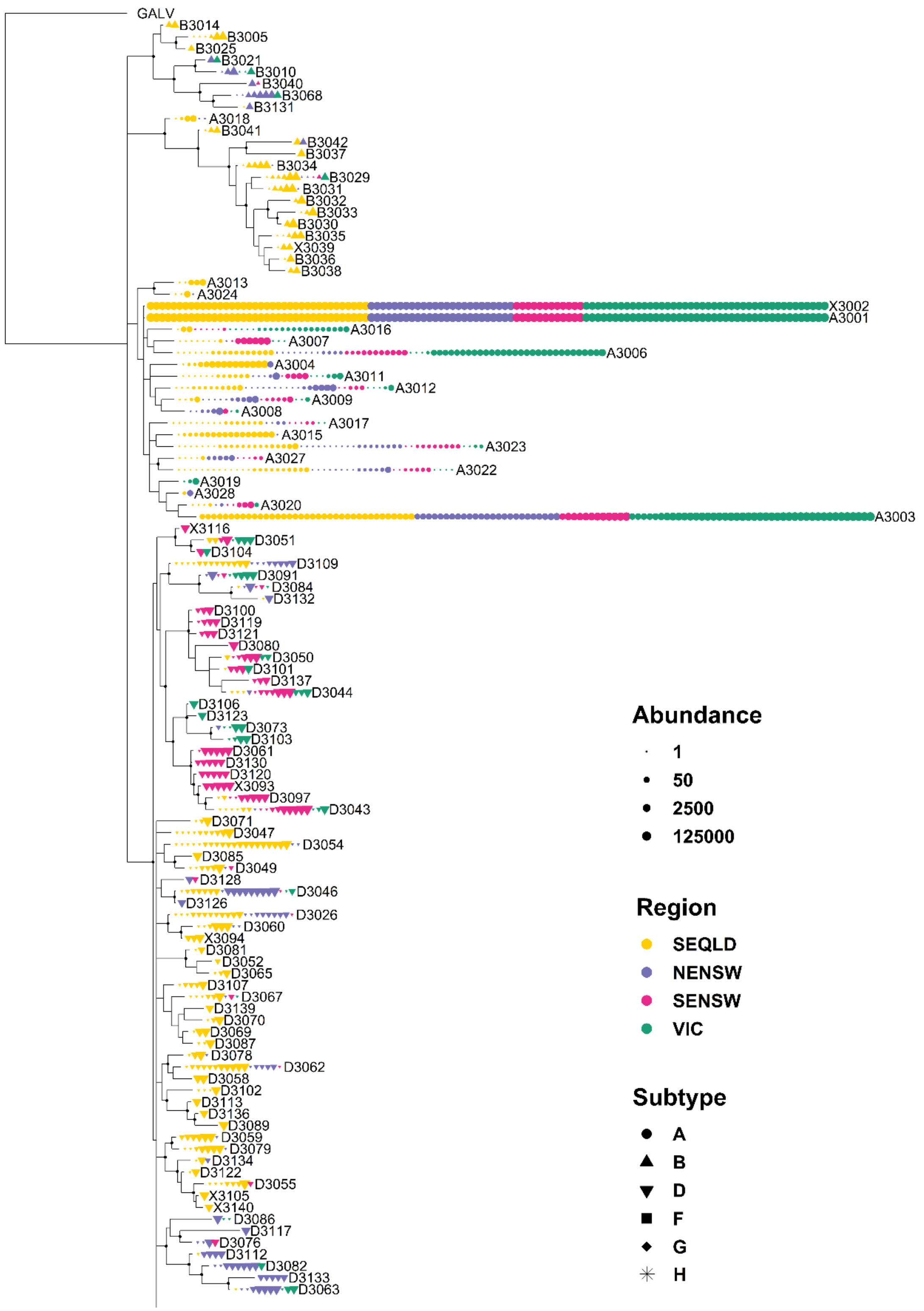

Supplementary Figure 4. Bayesian phylogenetic tree (part 1)

MOLECULAR ECOLOGY

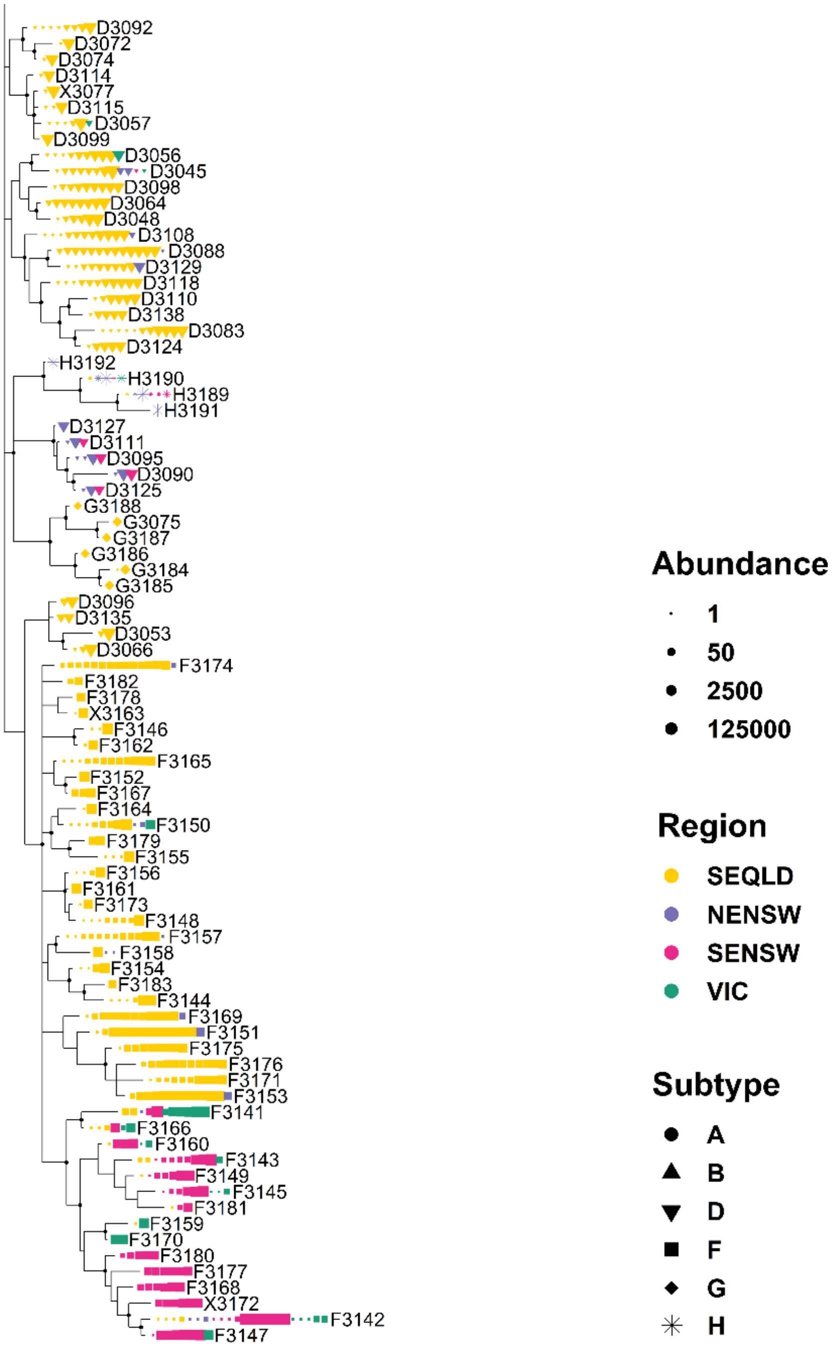

Supplementary Figure 4. Bayesian based phylogenetic tree (produced using MrBayes) of KoRV OTUs detected across Australia. Nodes with consensus support values greater than 90% are marked with a black point. Each branch represents a detected KoRV OTU, with individual koalas possessing that OTU represented next to the branch by a symbol. Symbols indicate the subtype of the OTU (shape), the region that koala is from (colour) and the abundance of this OTU's sequence reads from that koala (size). OTU labels starting with an X indicate defective *env* sequences.

MOLECULAR ECOLOGY

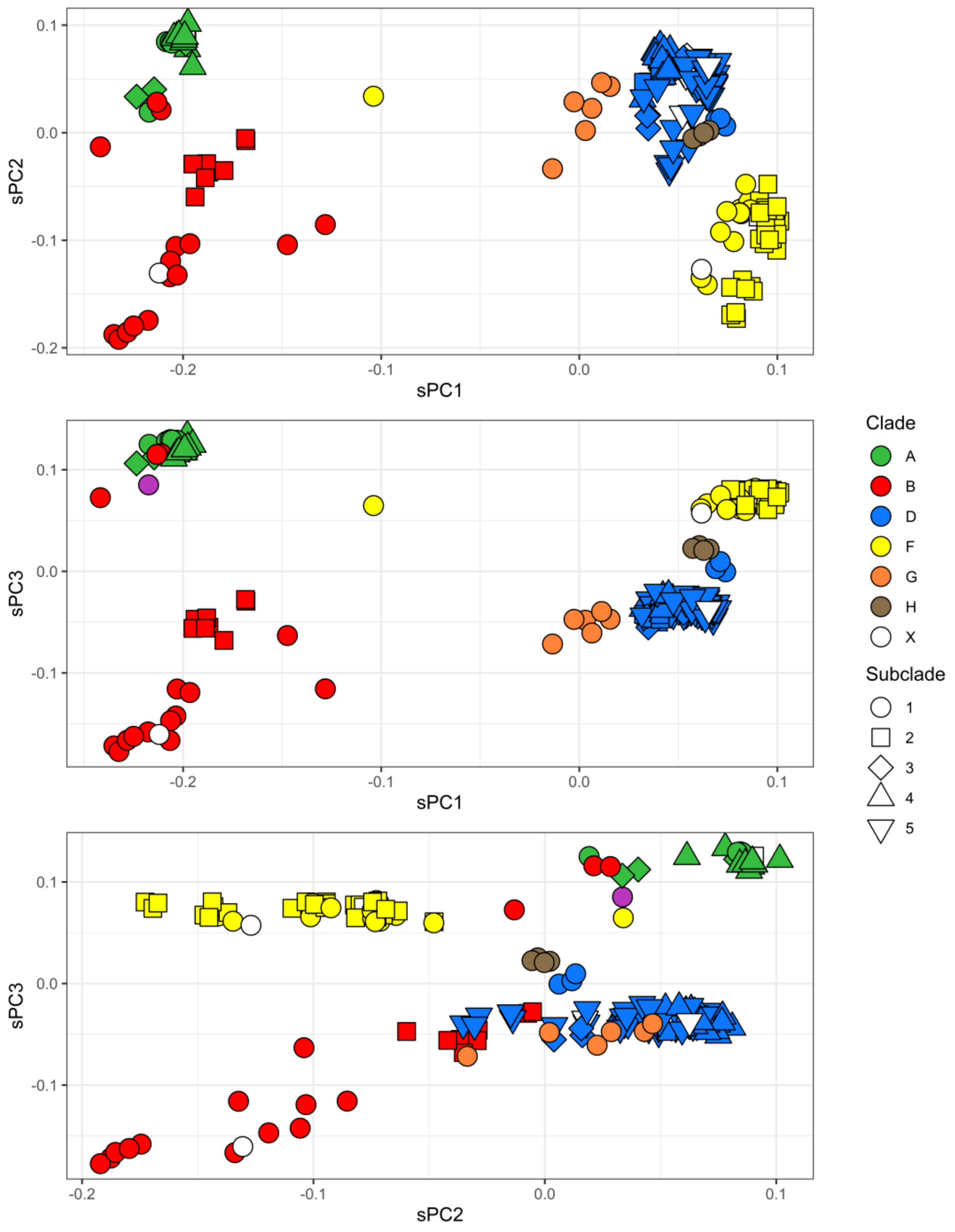

Supplementary Figure 5. Principal components analysis (PCA) applied to KoRV OTU sequence data. OTUs are coloured according to their subtype (X = defective, GALV = purple) while shapes show the subclade of the OTU as determined from the maximum likelihood phylogenetic tree presented in Figure 4. PCA was carried out using the script from: https://www.nature.com/articles/s41598-019-55253-0, based on the work from: Konishi, T., Matsukuma, S., Fuji, H. et al. Principal Component Analysis applied directly to Sequence Matrix. Sci Rep 9, 19297 (2019). https://doi.org/10.1038/s41598-019-55253-0.

MOLECULAR ECOLOGY

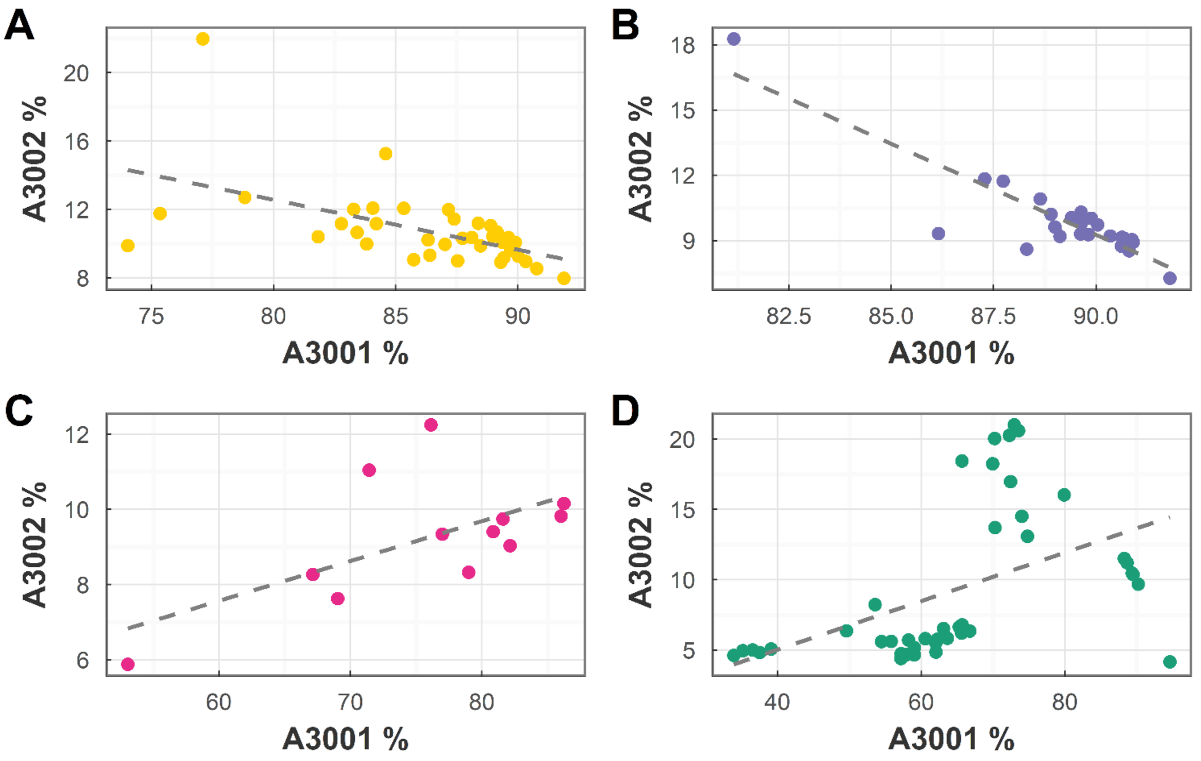

Supplementary Figure 6. Correlation between the abundance of A3001 and A3002 detected in each koala in (A) South East Queensland (SEQLD), (B) North East New South Wales (NENSW), (C) South East New South Wales (SENSW) and (D) Victoria (VIC). Regression axis labels indicate the percent of reads attributed to each OTU. Regression equations were (A) SEQLD: A3002 = – 0.29 A3001 + 0.36 (R^2 = 29%, p = 0.0005), (B) NENSW: A3002 = – 0.84 A3001 + 0.84 (R^2 = 78%, p = 4.6 × 10^{-9}), (C) SENSW: A3002 = 0.11 A3001 + 0.01 (R^2 = 37%, p = 0.035) and (D) VIC: A3002 = 0.17 A3001 – 0.02 (R^2 = 23%, p = 0.001).

MOLECULAR ECOLOGY

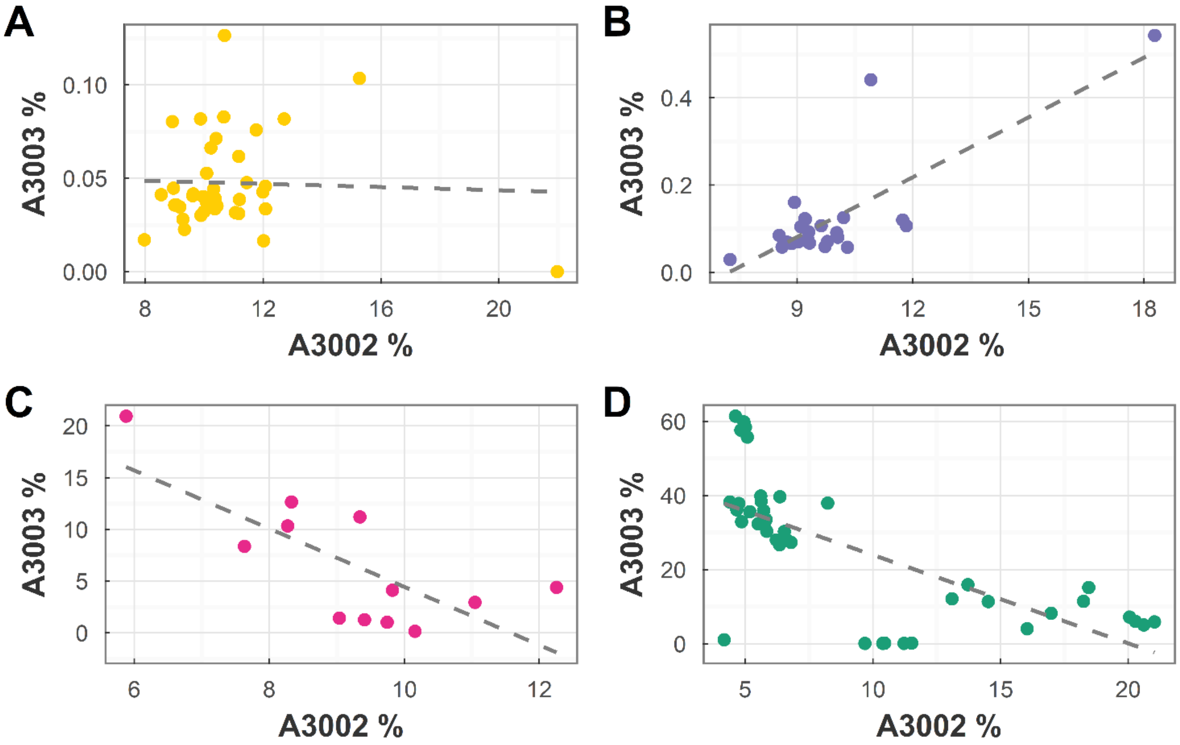

Supplementary Figure 7. Correlation between the abundance of A3002 and A3003 detected in each koala in (A) South East Queensland (SEQLD), (B) North East New South Wales (NENSW), (C) South East New South Wales (SENSW) and (D) Victoria (VIC). Regression axis labels indicate the percent of reads attributed to each OTU. Regression equations were (A) SEQLD: A3003 = – 0.0004 A3002 + 0.0005 (R^2 = 0.1%, p = 0.82), (B) NENSW: A3003 = 0.004 A3002 + 0.003 (R^2 = 62%, p = 2.8 × 10^{-6}), (C) SENSW: A3003 = – 2.8 A3002 + 0.33 (R^2 = 54%, p = 0.006) and (D) VIC: A3003 = – 2.34 A3002 – 0.48 (R^2 = 44%, p = 2.1 × 10^{-7}).
